# Oil Characterization, Molecular Modeling and Process Simulation in Petroleum Research and Industry, Opportunities, Challenges and Perspectives

**DOI:** 10.3390/molecules31101645

**Published:** 2026-05-13

**Authors:** Svetlin Vasilev, Dicho Stratiev, Ivelina Shishkova, Rosen Dinkov, Dobromir Yordanov, Tamer M. M. Abdellatief, Sotir Sotirov, Evdokia Sotirova, Aleksandar Dimitrov, Vania Georgieva, Tania Pencheva, Krassimir Atanassov, Radoslava Nikolova

**Affiliations:** 1 University Prof. Dr. Assen Zlatarov, Professor Yakimov 1, 8010 Burgas, Bulgaria; svetlyov@mail.bg (S.V.); ssotirov@btu.bg (S.S.); adimitrov@uniburgas.bg (A.D.); radoslava_nikolova@btu.bg (R.N.); 2 LUKOIL Neftohim Burgas, 8104 Burgas, Bulgariadinkov.rosen.k@neftochim.bg (R.D.); 3 Institute of Biophysics and Biomedical Engineering, Bulgarian Academy of Sciences, Georgi Bonchev 105, 1113 Sofia, Bulgaria; tania.pencheva@biomed.bas.bg (T.P.);; 4 Sustainable Energy & Power Systems Research Centre, The Research Institute of Sciences and Engineering (RISE), University of Sharjah, Sharjah 27272, United Arab Emirates; 5 Chemical Engineering Department, Faculty of Engineering, Minia University, El-Minia 61715, Egypt

**Keywords:** petroleum, oil characterization, molecular modeling, process simulation, refining

## Abstract

Petroleum is a valuable mineral resource from which base fuels and innumerable materials are produced. Its optimized utilization requires a deeper understanding of its chemistry, that can be obtained by improved characterization and molecular modeling approaches. Since the introduction of the molecular modeling strategy in 1989 up to now the number of publications has increased exponentially, but a comprehensive review, that tracks the evolution in the development of molecular modeling, based on improved oil characterization methods, and process simulation in petroleum research and industry, has not appeared yet. For that reason 459 published studies, dedicated to the progress in petroleum characterization methods, which support the advance in molecular reconstitution, and to process simulation in petroleum refining, found in Scopus, American Chemical Society (ACS), Wiley, Springer, Taylor and Francis, MDPI, Google Scholar and others for the period 1989–2025 and the beginning of 2026, were reviewed. From virtual oil molecules used in the pioneering studies of molecular modeling the advance in the semi-quantitative characterization of petroleum enabled the molecular models of petroleum to become molecular-characterization-informed models. The advance in the optimization algorithms together with the progress of the hardware allowed 423-fold reduction in computation time, that enabled involvement of the molecular reconstruction models in refining process models and optimizing refining performance. The progress achieved in the molecular reconstitution of petroleum allows its application in crude oil selection in petroleum refining and for geochemistry studies.

## 1. Introduction

Petroleum provides the energy required for modern society’s transport systems, and it ensures the fundamental chemical building blocks, known as petrochemicals, from which base innumerable materials are produced [1,2,3,4]. Without it, the economy of contemporary humanity would be completely blocked due to a non-functioning transportation system. An illustration of the big influence the petroleum industry has on society’s development is the established very strong correlation between daily volume of crude oil refined in each region and GDP (Pearson correlation coefficient R = 0.94) [5]. Considering its paramount importance for advancing humankind the efficient utilization of this valuable mineral resource is a question of special care.

Crude oil is perhaps the most complex mixture in the world in terms of the number of chemically diverse substances and the concentration range in which they exist [6,7,8]. That is why oil characterization and process simulation in petroleum refining are highly challenging tasks [9,10,11,12]. The characterization of crude oils and petroleum fractions is an important step in process simulation applied in the petroleum industry [13,14]. The more comprehensive and accurate the oil characterization, the better and more adequate the simulation of the petroleum refining process [15,16,17]. In modern petroleum science various advanced analytical techniques have appeared recently which allow a more comprehensive characterization of petroleum and its derivatives [18,19,20,21]. Among the modern analytical methods used in petroleum science lately those based on mass spectroscopy are most prevalent [6,8,22,23,24,25,26,27,28,29,30,31], followed by the combination of other techniques such as high-performance liquid chromatography (HPLC) [20], silica sulfuric acid sulfonation [32], hetero-medium-pressure liquid chromatography [33], gas chromatography [34] with mass spectroscopy, and nuclear magnetic resonance (NMR) [35,36,37]. These advanced analytical techniques have enriched the knowledge of petroleum chemistry, allowing characterization of oils at the molecular level [38,39,40,41]. Along with the progress made in petroleum characterization through advanced analytical methods, another approach has been developed in parallel that also provides molecular details of petroleum composition, coined as molecular modeling [42,43,44,45,46], which enabled the application of molecular refining [47,48,49]. Molecular modeling of petroleum is a computer simulation based on petroleum characterization data and applying various computational techniques to generate individual molecules, predict their properties, construct a composition model, create a reaction network, and build a kinetic model [50]. It can be viewed as a bridge between petroleum chemistry and computer simulation, and as such can be considered a part of mathematical chemistry. In addition to molecular modeling two other modeling methods are typically used in petroleum industry process simulation: pseudo-component generation [11,51,52] and chemometrics combined with partial least squares (PLS) and machine learning [13,19,53,54] methods. Pseudo-components are generated from the curves of true boiling point (TBP) distillation and specific gravity as narrow fractions whose boiling point and specific gravity are defined as the average boiling point and specific gravity of the distilled fraction. They are considered as substitutes for the individual components making up petroleum fluid with the relevant characteristics calculated from their TBP temperature [55]. Chemometrics is considered a chemical discipline that applies statistical and mathematical methods based on formal logic to extract relevant chemical information through the analysis of chemical data [19]. Machine learning (ML) is considered a part of artificial intelligence (AI) that applies training algorithms to huge datasets to develop models that can predict outcomes, classify information, and make decisions based on patterns and relationships in the data [56].

Although the pseudo-component generation approach has proven useful in simulating fractionation processes [55,57,58], the reaction kinetics cannot be well represented in this way because pseudo-components do not contain the necessary detailed information, such as individual structural parameters or specific group compositions in the fractions [48]. Molecular modeling is a more comprehensive approach that allows for property calculations and development of kinetic models at the molecular level using a consistent molecular grouping strategy [48]. Chemometric methods have found wide application in oil refining practice, but they require a large amount of data for model development and calibration, which takes a lot of time and analytical resources [19].

Petroleum molecular modeling over the years has applied different techniques such as stochastic reconstruction (SR) [59,60,61], entropy maximization (REM) [61,62,63], molecular type homologous series (MTHS) [64,65,66], hybrid structural unit and bond-electron matrix (SU-BEM) [67,68,69] as well as structure-oriented lumping (SOL) and group contribution methods [70,71] and machine learning methods for modeling petroleum fluids [72,73,74,75,76] and their processing [77,78,79,80]. The various petroleum molecular modeling approaches, also called reconstitution methods, provide detailed molecular compositions employing the techniques mentioned above on the base of diverse analytical characterization procedures. These reconstitution methods allow, in most cases, the use of actual chemical compounds, which can be analyzed for their structure and have their properties calculated from experimental data or predicted using group contribution methods [55,81,82]. The use of actual chemical compounds or hypothetical compounds, where the molecular structure is described using structural lumps, allows the researcher to identify and describe the possible reactions each component can undergo. This is a more direct approach in simulating the behavior of the petroleum and petroleum fractions than the use of pseudo-components, which represent an indefinite number of pure components within a narrow boiling point range, each with different structures and properties. This in turn will result in more accurate prediction of the simulation model’s phase equilibria, thermodynamic properties and unit operation performance prediction and sizing [9,10,55]. Regardless of the progress in the field of the characterization of petroleum fractions, these new methods remain largely unincorporated in the most used commercial process simulators (Aspen HYSYS [83], Aspen Plus [83], ChemCAD [83], WinSin Design II [84], Aveva Pro/II [84], ProMax [84], Honeywell UniSim [84], Schlumberger VMG Symmetry [85]), the open-source DWSIM [86,87,88] and the free COCO Simulator [87,88,89]. This requires the user of these process simulators to occasionally run the pseudo-component generation routines outside of the process simulator environment and manually enter the generated pseudo-components, which may contain individual structural parameters or specific group compositions [89] obtained using different molecular modeling approaches, as user-added components. The pseudo-components generated on the base of data for density and distillation curves, which represent a simplified approximation of petroleum composition, have found a wide application in fractionation simulation studies [90,91,92] because of the good balance between accuracy and computational efficiency. Liu et al. [10], however, reported that the pseudo-components generated in this way suffer from a lower accuracy in simulation of dynamic changes, which occur during distillation. Furthermore, pseudo-components, which lack molecular-level details, cannot accurately represent the kinetics of reactions in petroleum refining processes [48]. The molecular-level details of petroleum fluids can be modeled using the molecular reconstitution approach generating digital oil model that enables more accurate simulation of processes in the petroleum industry and optimization of their performance [93,94,95,96].

The first steps towards molecular modeling of petroleum can be deemed the pioneering works of Savage and Klein in 1980s [97,98] who linked the reaction pathways and kinetics of asphaltene thermolysis with the molecular structural characteristics of asphaltenes. Later Liguras and Allen [99,100] in 1989 first introduced a set of predefined molecules to build a model for molecular-level reconstruction of catalytic cracking reactions of hydrocarbon mixtures, and the molecular modeling method has undergone considerable progress by applying various techniques and approaches [43,48,49,101].

Ren et al. [43] summarized the progress achieved in the field of petroleum reconstitution for the period 1989–2018. They reviewed and collated the methods for oil reconstruction models: SR, SOL, MTHS, REM, SR-REM and state space representation method.

Wang et al. [47] in 2023 systematically generalized the theoretical essence of SOL, its different approaches and the directions of the future elaboration of this method of molecular modeling of petroleum.

Chen et al. [49] in 2024 reviewed the advance in the molecular-level kinetic models applied in oil refining conversion processes. They discerned the different modeling techniques for constructing the molecular-level kinetic models as the single event kinetic model, BEM, SOL method, SU-BEM, and MTHS matrix. It was concluded that the application of molecular-level kinetic models in petroleum refining is hampered by high computational costs, lack of information on catalysts, and insufficient data on industrial stream characteristics [49]. They suggest that these challenges can be overcome by developing a rapid analytical method for industrial streams that provides the molecular composition of oils; creating catalyst descriptors to be included in kinetic models at the molecular level; introducing a new strategy for simplifying the reaction network and solving models; and further improving computational efficiency.

Wang et al. [48] in 2025 developed a simulation model based on the SOL molecular modeling framework of the whole petroleum refining process that transforms the crude oil feedstock into finished marketable products. The model involved 12 refinery units and molecular-level models were constructed for the processes: atmospheric and vacuum distillation, delayed coking, residue hydroprocessing, vacuum gas oil (VGO) hydroprocessing, fluid catalytic cracking (FCC), hydrocracking, diesel hydrotreating, jet fuel hydrotreating, gasoline hydrotreating, catalytic reforming, aromatic extraction, gasoline etherification, naphtha hydrotreating, alkylation, isomerization, methyl tert-butyl ether (MTBE) production, gas fractionation, and hydrogen production. They simulated the operations of three refineries, A, B, and C, using the constructed molecular-level models. The operations of Refinery A were simulated to process crude oil, whose reconstructed molecular model contained 8030 molecules. The operations of Refinery B were simulated to process crude oil, whose reconstructed molecular model contained 7792 molecules. The operations of Refinery C were simulated to process crude oil, whose reconstructed molecular model contained 12,465 molecules. The reconstructed molecular models of the chemical composition of the crude oils simulated for processing in the three refineries studied were built based on the information from the crude oil evaluation reports using ASTM standards. The authors summarize that they have created a method for analyzing the molecular composition of crude oil, but unfortunately, in their study they do not disclose how this method was developed and which properties of the crude oil, measured according to ASTM standards, were used. They properly note that the number of molecules in the reconstructed molecular model of crude oil affects the accuracy of the simulated results of the overall process model. However, they do not discuss why the numbers of molecules involved in the reconstructed chemical compositions of the crude oils for the three studied refinery configurations are different and whether this distinction is a result of diverse crude oil evaluation reports.

Different studies have reported that the number of molecules that need to be included in a molecular-level model for oil reconstitution can vary. For example, Trauth et al. [102] determined that 10,000 molecules were enough to simulate the properties of vacuum residue, such as H/C ratio, molecular weight, and SARA composition. Jaffe et al. [103] deduced that ~1500 composite molecules are adequate to encompass the compositional diversity established in petroleum residue fractions. Researchers from the French Institute of Petroleum (IFP), who worked on vacuum residue reconstitution [61,104,105,106,107,108], discovered that 5000 vacuum residue molecules are the optimal balance between the required CPU time and the accuracy of the residue representation. Recent advances in computer hardware and algorithms [109,110,111,112,113] allowed improvement in molecular modeling by increasing the number of representative molecules of the modeled oil. All molecules in the reconstructed models of Trauth et al. [102], Jaffe et al. [103], and the IFP researchers [104,105,106,107,108] are virtual and the molecular content is determined without reference to molecular-level characterization information. The progress attained in the analytical methods allowed a great deal of heavy oil molecules to be recognized and quantified. For example, Li et al. [38] detected and quantified more than 7000 molecules in a sample of heavy FCC slurry oil (fraction boiling between 280 and 725 °C) using high-resolution Orbitrap mass spectrometry. Researchers have gained from the improvement in the recognition of heavy oil molecules obtained by advanced analytical techniques to develop molecular compositional models [113,114,115]. Zhang et al. [113] built a molecular compositional model for heavy oil and confronted it with high-resolution mass spectrometry (HRMS) data, showing that the molecular distribution was in good agreement with the carbon number and DBE scope obtained from the spectrometry data. Alvarez-Maymutov et al. [114] developed a model of the molecular composition of the vacuum residue, that involved 5000–10,000 molecules, and the model results were consistent with time-resolved fluorescence depolarization and mass spectrometry in terms of molecular distribution. Guan et al. [115] engineered a molecular-level reconstitution model of heavy petroleum represented by 12,734 molecules availing HRMS data as validation, and the precision of molecular fraction distributions has been enhanced. Guan et al. [116] introduced an HRMS-informed molecular reconstitution approach, and the molecular composition of four VGOs was accurately reconstructed. They found that HRMS detected about 8000~10,000 molecules and almost all detectable molecules were included in their VGO molecular library that consisted of 12,138 molecules.

**Figure 1:**
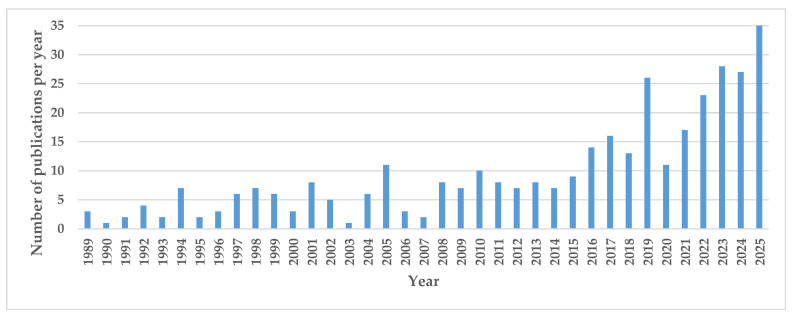
Number of publications per year related to molecular modeling of chemical compositions of petroleum and oil fractions (data were extracted from Scopus, American Chemical Society (ACS), Wiley, Springer, Taylor and Francis, MDPI, Google Scholar and others, included in Table 1).

Advances in molecular modeling of petroleum over the years have allowed for greater incorporation of petrochemical knowledge into the design, operation, and optimization of petroleum refining processes. This can explain the exponential increase in the number of studies devoted to oil reconstitution, as illustrated in Figure 1.

The progress in oil characterization methods along with hardware improvements and the use of enhanced algorithms, which significantly reduce the computational time, allowed the number of representative molecules in the molecular model of petroleum chemical composition to be increased from about 10,000 [102] to 400,000 [113] in a period of 20 years, that in turn enhanced precision of the process models [48].

Over the years, significant progress has been made in the molecular reconstitution of petroleum, as reflected in recent review articles [43,47,49]. However, the detailed review by Ren et al. [43] summarizes the progress made in molecular reconstruction of petroleum fractions up to 2018, which covers 187 publications out of 356, which were issued in the period 1989–2025 (see Table 1). The review by Wang et al. [47] summarizes only the progress made in the SOL method, while the review by Chen et al. [49] focuses mainly on the construction of kinetic models at the molecular level for petroleum refining. Therefore, it is necessary to summarize further improvements in the methods for petroleum characterization and the modeling techniques and algorithms applied in molecular reconstruction of petroleum studies conducted after 2018 in order to track the progress made and identify existing opportunities, challenges, and future prospects for the application of molecular-level models in simulating processes used in petroleum refining practice, which is the goal of the present study.

## 2. Molecular Reconstruction Models of Petroleum Fluids and Oil Characterization Methods Supporting Their Progress

The literature search in the databases of Scopus, ACS, Wiley, Springer, Taylor and Francis and Google Scholar revealed the presence of 356 publications (articles, PhD theses, chapter of books, books, and patents) for the period 1989–2025 and the beginning of 2026. Table 1 epitomizes the publications that appeared each year for the period 1989–2025, and in the beginning of 2026, and investigated the molecular modeling of petroleum and oil fractions.

Among the discovered publications colligated in Table 1 the number of articles is 317, that of PhD theses is 25, chapters of books—4, books—7, and patents—4. The organizations which have been working in this field are ExxonMobil [103,122,135,150], Shell [48], Dow Chemical [48], French Institute of Petroleum (IFP) [61,72,104,105,106,107,108,169,172,173,174,231,250,251,288], PetroChina Company Limited [403], Aspen Technology [13], University of Delaware [123,144,164], University of Manchester [151,152,177,203,207,243,256,276], Rutgers University [176,200,218], Istanbul University [74], Ghent University [72,191,197,209,226], Boreskov Institute of Catalysis [321,376,378], Institute of Organic Chemistry, Russian Academy of Sciences [207], etc. Various software packages, such as Structural Molecular Generation (SMOG) (Institute of Organic Chemistry, Russian Academy of Sciences) [207], KMT, KME [193], Composition Model Editor (CME) [217,235], Interactive Network Generator (INGen) [200,235], Network Generator (NetGen), RMG, Genesys [126,226,227,249,419], and others, have been developed and employed in diverse oil molecular modeling studies.

**Figure 2:**
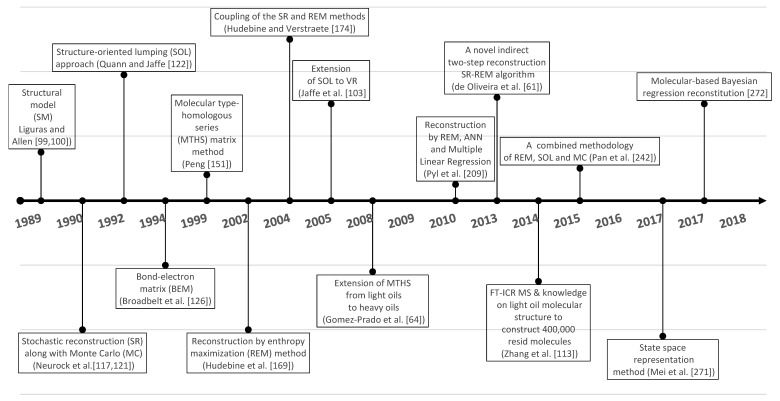
Milestones in the development of oil molecular reconstitution models for the period 1989–2018 [61,64,99,100,103,113,117,121,122,126,151,169,174,209,242,271,272]. (This is an upgraded and modified version of Figure 1 in the research by Ren et al. [43]).

Ren et al. [43] have already analyzed the progress in the development of molecular reconstruction modeling techniques for petroleum for the period 1989–2018, but some details that might be of interest to petroleum and refining engineers were not mentioned, which will be discussed in this review. Figure 2 generalizes the main stages in the development of molecular models of petroleum oils for the period 1989–2018. It is more detailed and upgraded compared to that reported in Figure 1 in the work of Ren et al. [43]. During the period 1989–2018, the foundations of molecular management technology for oil refining were laid. Its aim was to study the chemistry of petroleum in as much depth as possible, reaching the molecular level, and on this basis to try to better understand the transformations that occur during the various refining processes, attempting to control these transformations and steer them in the desired direction. Four molecular modeling frameworks were introduced for that period: SR, SOL, bond-electron matrix (BEM), and MTHS, the essence of which will be discussed in more detail in Section 2.1. It was found that the accuracy of the model’s predictions depends on the number of molecules involved in the oil reconstitution process and that the calculation time increases with the molecular number [130]. The greater the number of molecules, the higher the accuracy of the predictions and the longer the computation time [130,137]. Therefore, the right balance between acceptable accuracy and reasonable computation time was searched for [61,104,137]. The progress in the hardware and the algorithms used enabled increasing the number of molecules involved in the petroleum reconstitution process along with shortening the reckoning time. For example, in 1997 the computation time to reconstruct the molecular composition of vacuum residue by the SR method took 72 h [137], while in 2014 it required 4 h [113]. Oil molecular reconstitution methods have become an integral part of the simulation models of refining processes such as naphtha reforming, naphtha hydrotreating, gas oil hydroprocessing, FCC, naphtha pyrolysis, delayed coking, oil fractionation, gasoline blending, etc. [187,193].

The application of the modeling frameworks to simulate refining processes like residue FCC (SOL) [267] and residue pyrolysis (BEM) [247] showed a remarkable difference for the generation of reaction networks: 25–30 h for SOL versus 30 s for BEM, underlining the advantage of the BEM technique to model refining processes. Undoubtedly, the depth that these models provide can be used to improve process design, process operation and performance, and catalyst design and to increase the efficiency of utilizing the potential of petroleum feedstocks.

As can be seen from the data in Figure 1, the number of publications for the period 1989–2018 can be averaged at 6 per year, while the number of publications for the period 2019–2025 can be averaged at 23 per year, which indicates a significantly increased interest in molecular modeling of petroleum and its application in refining process simulation.

Irrespective of the publication of seven books [187,196,253,260,286,305,306], 23 PhD theses [74,121,123,124,143,144,151,152,164,172,176,177,200,203,206,207,218,231,234,256,276,295,302], three chapters of books [156,255,376], four patents [245,282,288,403] and 317 articles (see Table 1), Zhang et al. [313] concluded in 2020 that the molecular modeling of petroleum is still in its incipiency. They noted that there are still areas where further progress is needed. These areas are the provision of accurate qualitative and quantitative analysis of all types of molecules and supramolecular systems in heavy petroleum fractions. There are still debates about the structure of most complex components of crude oil—asphaltenes. Oliver Mullins’ research group argues that “island” or “continental” structures are the predominant architecture of asphaltenes [420,421,422,423,424]. Whereas the studies of the research group of Ryan Rodgers [425,426,427,428] showed that two structures of asphaltenes, “island” and “archipelago”, coexist and the prevalence of either of them is dependent on the sample [427]. The research group of Murray Gray investigating both thermal cracking and catalytic hydroconversion indubitably proved the existence of archipelago structures of asphaltenes [429,430,431]. The understanding of the mechanism of formation of supramolecular systems in heavy oils is also controversial, with proponents of the “island” model favoring the π–π stacking of aromatic rings [423,432], while Gray’s research group considers that the supramolecular systems are a result of cooperative binding by Brønsted acid–base interactions, hydrogen bonding, metal coordination complexes, and interactions between cylcoalkyl and alkyl groups [433,434,435]. The complexity of asphaltenes is so great that it seems that no concept is definite. Thus, molecular-level representation of asphaltenes from a certain crude oil may completely misrepresent that of a different crude oil, that makes the molecular modeling of various heavy oils very challenging task.

Developing reliable simplification rules that reduce computational costs while adequately describing the behavior of petroleum molecules during separation and transformation processes in petroleum refining is another challenge that needs to be addressed.

It can be concluded that further development of molecular petroleum management technology can be achieved by following two directions: (1) introduction of accessible analytical methods that provide qualitative and quantitative characterization of petroleum molecules; (2) construction of more accurate and faster models of molecular composition and processes based on more mechanistic information and adequate simplification rules.

**Figure 3:**
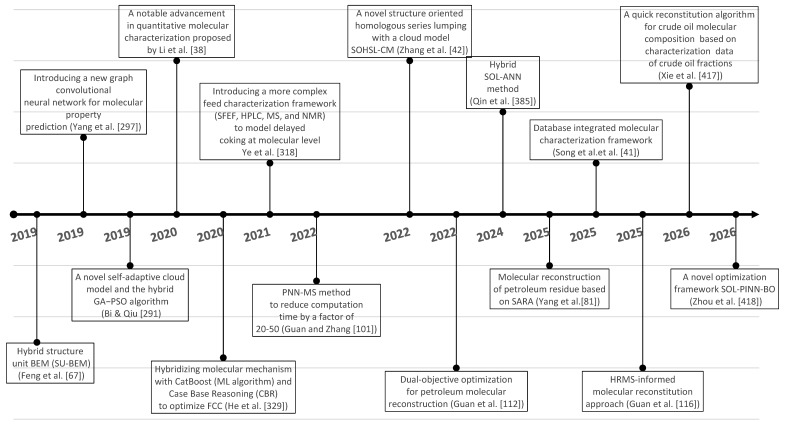
Major improvements in the molecular modeling of petroleum composition for the period 2019 ─ 2025 and early 2026 [38,41,42,67,81,101,112,116,291,297,318,329,385,417,418].

Figure 3 shows the main advances in analytical procedures and molecular modeling technology for petroleum for the period 2019–2025 and early 2026, related to the emergence of new methodologies and the improvement of existing ones, leading to higher accuracy in predicting petroleum properties and shorter calculation time.

During the period under review, 2019–2025 and the beginning of 2026, a tremendous improvement in the reconstitution algorithms was observed, leading to a substantial reduction of the computation time (from the best computation time of the period 1989–2018 of 4 h for VR reconstitution [113] to 604 [417] (24 times)) without any penalty in the accuracy of prediction. Song et al. [41] reported even shorter computation time for the reconstruction of petroleum residue molecular composition of between 3 and 36 s with the algorithm proposed by them.

In the period 2019–2025, several diverse hybrid modeling techniques were applied: SU-BEM [67], MAG-SOL [71] and SOL-ANN [395], transparent chemical process assisted by artificial intelligence [101], ML-REM [416] and others, which combined the strengths of different methods to make the molecular modeling process more efficient in terms of calculation speed, modeling accuracy and robustness and stability of the algorithms used. Advances in petroleum characterization methods reported in the research of Quan Shi’s research group [30,38,39,436,437], based on ultrahigh-resolution mass spectroscopy combined with derivatization methods, allowed the quantification of up to 10,000 petroleum molecules and the incorporation of these data into a dual-objective optimization framework, thus establishing a new HRMS-based molecular reconstitution approach [116]. Thus, in the construction of petroleum compositional models it becomes possible to include as many of the detectable molecules as possible and make the model much closer to the actual petroleum molecular composition. This is in contrast to the models relying only on virtual molecules whose molecular content was determined without reference to molecular-level characterization information in the pioneering studies of Klein’s research group [102,127,137] and Verstraete’s research group [61,173,174,210]. Besides petroleum fractions, the molecular composition of whole crude oil was modeled in the studies of Liu et al. [9], Zhang et al. [42], Zhou et al. [336,386,401], Chen et al. [412] and Xie et al. [417], considering that the composition of whole crude oil is much more complex than that of petroleum fractions in terms of a broader distribution of boiling point, carbon number, molecular weight and molecular structures of hydrocarbons and heteroatoms. The molecular models of oil chemical compositions were incorporated in process models to predict performance of oil refining processes: crude distillation (Liu et al. [9,10]), FCC [68,339,365,366,385,395], hydrocracking [370,372], naphtha reforming [369,379,394,400]. Despite the progress achieved in both modeling techniques and petroleum characterization methods the fraction of petroleum most difficult to characterize and model—vacuum residue—is still difficult to model using quantitative molecular characterization information and relies mainly on virtual molecule representations. The reason lies in the complexity of the semi-quantitative analysis of molecular composition. The samples need to be pretreated several times before analyzing them by HRMS and then integrated with a quantitative algorithm to obtain the results of semi-quantitative analysis of molecular composition [436]. The more detailed the characterization procedure, the more accurate the molecular reconstruction process, which was evident from the comparison between the reconstitution results of petroleum residue reported in the research of Ye et al. [318] and Tian et al. [225]. However, detailed characterization of petroleum residues is very time-consuming and expensive to implement on an industrial scale. For this reason, the study by Yang et al. [81] represents an interesting and promising alternative to detailed characterization of residual oils using SARA fraction and elemental composition and simulated distillation to reconstruct the molecular composition of the oil. Further studies are needed to validate the method of Yang et al. [81] for vacuum residues with more than 7 wt.% asphaltene content, since asphaltenes as mentioned earlier are the most complex fraction of crude oil and the most difficult to model.

**Figure 4:**
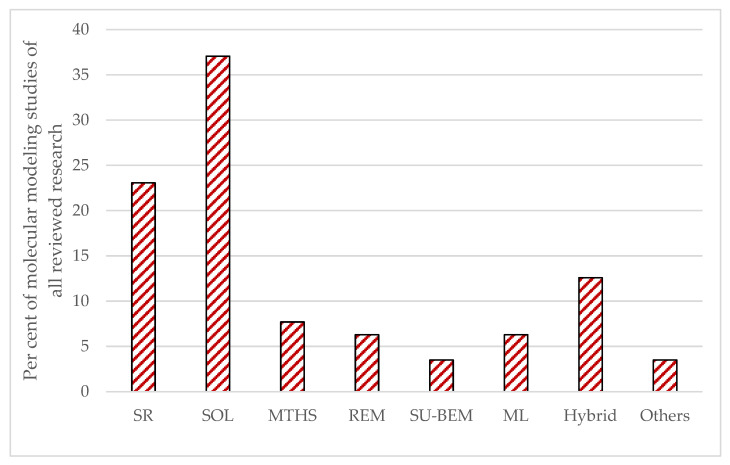
Distribution of molecular modeling frameworks used in the research, published between 1989 and 2025.

The molecular modeling frameworks utilized in the various reconstitution investigations of petroleum and its fractions over the reviewed period of 1989–2025 and beginning of 2026 showed that the SOL method was the most frequently explored, following by SR and hybrid methods, with other methods corresponding to about one third of all molecular reconstruction studies (Figure 4). It should be noted here that the hybrid and machine learning approaches, which utilized the SR and SOL methods, were also added to SR and SOL frameworks. The remaining molecular modeling methods used in the reviewed studies accounted for less than 8% each.

### 2.1. Molecular Reconstitution Approaches

The key characteristics, advantages, and limitations of the main molecular reconstitution frameworks discussed in this review are summarized in Table 2.

Heavy oils are on one hand the largest petroleum resource and on the other hand the least valuable oil products. Their optimal utilization is vital for modern refining profitability. The data in Table 2 indicates that SR and SOL are the most suitable for molecular modeling of heavy oils. This can explain the predominant employment of these two frameworks over the other techniques, as most molecular modeling studies investigate heavy oils.

Concerning the accuracy and computational cost of molecular reconstitution models of petroleum, the performed studies showed that they depend on several factors. De Oliveira et al. [61] communicated that the hybrid SR-REM framework ensures higher prediction accuracy than SR alone when straight run vacuum residues are reconstituted. Yang et al. [81] demonstrated that the SOL-based method secures higher prediction accuracy than the SR framework when Venezuelan straight run vacuum residue is reconstructed. Therefore, one may conclude that the molecular modeling framework influences the accuracy of petroleum property prediction. Feng et al. [67] indicated in their research that the accuracy of molecular reconstruction of diesel fractions was higher than that of FCC slurry oil, suggesting that the nature of petroleum fluid also affects the accuracy of the molecular reconstitution process. The number of molecules was another factor impacting the accuracy of the molecular reconstitution process, with bigger numbers providing higher accuracy but unfortunately also higher computational cost [130]. The more detailed sophisticated characterization of petroleum fluid was also found to be a factor contributing to the higher accuracy of the molecular reconstitution [225,318]. The computational cost was observed to decrease with hardware improvement and the molecular modeling technique employed. For example, the SR method on an IBM RS/6000 computer (1997) needed 72 h to reconstruct vacuum residue and 48 h to reconstitute naphtha [137]. Whereas the MTHS method applied for reconstitution of light and heavy oils in 2025 required only between 3 and 36 s [41].

In reviewing publications since 2018, which represent about half of the publications on molecular modeling of petroleum, we noted some underestimation of the chemical nature of petroleum fluids being reconstructed. For example, Zhang et al. [42] in their study omitted to report the properties of the 42 crude oil sets that were modeled. In another study Zhang et al. [42] also did not communicate the characteristics of the 52 sets of crude oil data that were molecularly reconstructed using the SOL framework. Qin et al. [385], who constructed the SOL-ANN property prediction model of the FCC process of vacuum gas oil, also did not present any data about the FCC feed being reconstructed. Xie et al. [417] reported the TBP distillation characteristics of 22 crude oils that they reconstructed, showing a lack of naphtha fractions and in some cases a lack of middle distillates, while also reporting that naphtha and middle distillate reconstitutions are parts of the overall reconstruction process of the entire crude oil.

In the review papers of Ren et al. [43], Wang et al. [47] and Chen et al. [49] nothing has been commented about the origin and the specific characteristics of the oils being reconstructed. Mentioning the origin of the oil being reconstructed, as Campbell and Klein [137] (see Section 2.1.1) and de Oliveira et al. [61] (see Section 2.1.4) have done, is useful and valuable as some vacuum residues such as Vasconia (Colombia), Castilla (Colombia), Chichimene (Columbia) and Ku-Maloob-Zaap (Mexico), for example, cannot be processed by ebullated bed hydrocracking [438], while others like Maya (Mexico), for example, are challenging to process in ebullated bed vacuum residue hydrocracking [439] (conversion not higher than 40%), and Arabian light vacuum residue is easy to hydrocrack to high conversion levels (higher than 85%). The reasons for the intractability of the vacuum residues Vasconia, Castilla, Chichimene and Ku-Maloob-Zaap in hydroprocessing due to the appearance of loss of fluidization in the ebullated bed hydrocracking reactor [438] remain unclear. This can be considered a good subject for further investigation by the molecular modeling technologies for revelation of the root cause of this particular behavior of the mentioned vacuum residues. These facts made us pay attention in this review to the origin of the oils being molecularly reconstituted in the reviewed research.

Section 2.1.1, Section 2.1.2, Section 2.1.3, Section 2.1.4 and Section 2.1.5 present a more detailed summary of the reviewed studies using the different molecular modeling frameworks for petroleum for the period 1990–2025 and early 2026.

#### 2.1.1. Stochastic Reconstruction

**Figure 5:**
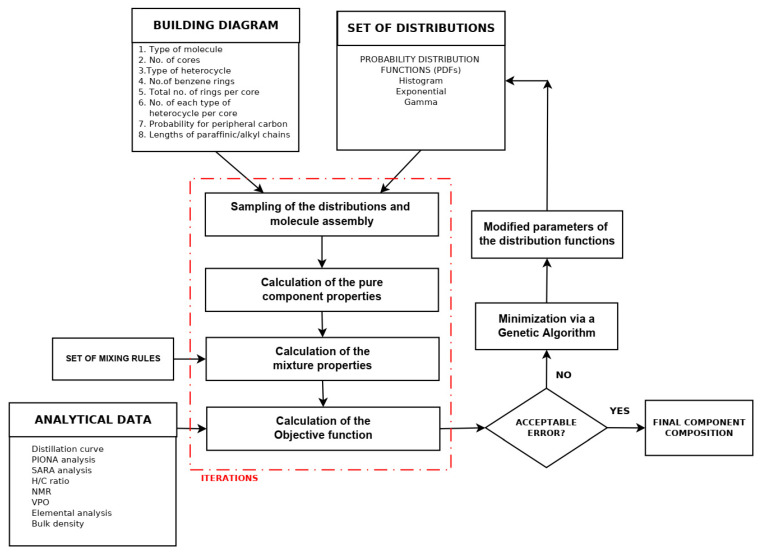
Block scheme of the SR method established by Klein’s research group (modified and upgraded from Hudebine and Verstraete [174]).

Neurock et al. [117], using analytical data from 1H NMR, vapor phase osmometry, and elemental analysis, transformed them into cumulative probability density functions (PDFs) for each structural attribute of asphaltene molecules, thereby constructing a stochastic model of 10,000 structures of an asphaltene feedstock undergoing hydroprocessing. The molecular details were acquired by Monte Carlo simulation. Neurock et al. [127] further refined the SR model to simulate molecular representation of complex hydrocarbon mixtures such as asphaltenes, heavy oil, and gas oil using a new Monte Carlo structural simulation approach. This simulation method consisted of four steps: (1) the creation of a chemical logic diagram, (2) the parametrization of cumulative probability functions for the structural attributes, (3) the stochastic sampling of these distributions, and (4) the transformation of this information into representative molecular structures [127]. The application of this simulation method to reconstruct the molecular composition of sour imported heavy gas oil (SIHGO) showed that total carbon distributions and SimDis curves were reproduced to within standard deviations of 11 wt.% and 20.8 °C. Underestimation of low boiling material and overestimation of high boiling material of SIHGO were observed, suggesting the appearance of molecules with untypically low and high molecular weights. Neurock et al. [127] deduced that these errors were most probably a result of the assumption of independent probability distributions and that they could be avoided by using a conditional PDF. The Monte Carlo simulation algorithm allowed easy reading of molecules, their important structural attributes, and the exact atomic configuration of each component. It presented a direct input for molecular reaction models and enabled the calculation of both global and molecular product properties as a function of reaction conditions [127]. Trauth et al. [102] additionally upgraded the SR method by uniting the analytical data from NMR, molecular weight by vapor pressure osmometry (VPO), and SARA analysis with an iterative stochastic modeling method to quantitatively represent the structure of petroleum residue. They approximated all probability density functions by the chi-square distribution that is a special case of the gamma distribution function. The molecular simulation of petroleum residue demonstrated an excellent fit of H/C atomic ratio, a good fit of saturate and aromatic content, and a fair fit of the molecular weight, but the asphaltene content and the simulated distillation characteristics were not very well simulated. The discrepancies between measured and simulated values of the simulated distillation characteristics and asphaltene content could be a result of inaccurate assumptions during molecule construction or the limited flexibility of the chi-square distribution. In summary, the ultimate molecular representation of any petroleum fraction by the SR method depends on the functional type of the oil mixture molecule (paraffin, aromatic, or naphthenic) and the parameter values of each PDF [130]. Petti et al. [130] observed that the CPU time linearly increases with the number of molecules in petroleum reconstruction models and that sample size had a considerable effect on the outcome of the optimization objective function both in terms of average and variability (standard deviation). They generalized that 10,000 molecules is the compromise between admissible accuracy of simulation and reasonable computation cost. Campbell and Klein [138] further refined the SR methodology by introducing a transformation of the basic analytical chemistry for petroleum oils into a small set of 20–30 representative molecules and extended the upper limit of the number of molecules to 100,000. They reported that the computer processor time required to determine the optimal PDF parameters on an IBM RS/6000 computer for petroleum residue reconstruction was about 72 h, while for naphtha it was 48 h [139]. Figure 5 shows a graphical representation of the methodology developed by Klein’s research group [138,187] using stochastic modeling of molecular structures and compositions of complex petroleum oils. It is worth noting here the observed differences in both properties and the resulting molecular reconstruction of two distinct vacuum residues—Arabian Light (Saudi Arabia) and Maya (Mexico)—reported in the study of Cambell and Klein [138].

Detected differences in molecular weight, sulfur content, aromatic protons, and other detailed structures accessible through NMR data between two studied vacuum residues suggest the existence of unique molecular structures in the two diverse vacuum residues. Therefore, to address the dissimilarity of these two vacuum residues a considerably larger set of molecular structures will be required, if a common optimal set of molecules should be used for the molecular reconstitution. The assertion by Law et al. [300] that petroleum geochemists believe that all crude oils are formed from the same types of compounds and that the differences between them are the result of varying concentrations of these compounds must be supported by a substantially larger number of molecular structures, as shown by the study by Cambell and Klein [137] to account for the great variability of crude oil compositions. In the 1990s Klein’s research group reached the conclusion that the chi-square distribution was suitable to model petroleum residue molecule attributes: paraffin chain length, number of rings in naphthenics, number of aromatic rings in aromatics and resins, number of naphthenic and thiophenic rings in aromatics and resins, number of sidechain sulfurs, and number of unit sheets in asphaltenes [202]. However, the sidechain length in naphthenics and aromatics and resins was modeled by the exponential distribution, and the γ distribution was applied to model the number of side chains in naphthenics and aromatics and resins [202]. More than a decade later (2009) Klein’s research group established that the replacement of chi-square distribution functions by γ distribution ones along with some improvements in the construction algorithm ameliorated the accuracy of molecular reconstruction models between 4 and 26 times for the petroleum residues Hondo (California, USA), Maya (Mexico), Arabian Light and Arabian Heavy (Saudi Arabia) [202]. Five years later (2014) Klein’s research group made further improvement to the SR framework used to reconstruct petroleum residues [113]. They availed the cognition of chemical structures of light petroleum oils and the data of detailed Fourier transform ion cyclotron resonance mass spectrometry (FT-ICR MS) of high molecular residue fragmentation products to create a library of 385 nuclei and two inter-core linkages and 194 side chains/inter-nuclear linkage structural blocks (approximately 600 attributes) [247]. All structural block members were fitted up from independent and dependent PDFs to construct about 400,000 molecules to cover all classes of compounds which are present in petroleum vacuum residue. The range of variation of molecular weight of constructed molecules was between 300 and 1800, while that of double bond equivalent (DBE) fluctuated between 0 and 60 and those of aromatic and naphthenic nuclei were around 20 and 6, respectively. The model was applied to reconstruct Venezuela petroleum vacuum residue. The reconstruction showed that density, SARA composition (aromatics and polars) and sulfur content were not accurately predicted. The poor prediction of density might be a result of the lack of application of any mixing rules. The unsatisfactory forecasting of the distribution of aromatic fraction (aromatics, resins, and asphaltenes) obviously comes from the missing information about the solubility of the different molecules falling within the distinct solubility classes. The computation time was 4 h on an i7 desktop computer with 8 GB of memory. To cope with the extremely large number of molecules comprising petroleum residues (between 100,000 and 400,000), when modeling their reactions, Klein’s research group introduced the concept of the attribute reaction model (ARM) [202]. It deals with the modeling of the reactions of the structural attributes, instead of the reconstructed molecules. The structural attributes keep the information from the analytical data and retain the molecular nature of the composition. The ARM concept was first applied to modeling thermal cracking of the petroleum residues Hondo, Maya, Arabian Light and Arabian Heavy [202]. It was found that conversion and product distribution were well predicted for Arabian Light and Maya, while those of Hondo and Arabian Heavy were not well forecasted. The accuracy of ARM conversion and product yield predictions was found to be in line with the exactness of molecular reconstruction. The lower the value of the objective function, i.e., the higher the accuracy of the reconstructed molecular model, the higher the prediction precision of the ARM [202]. The ARM concept was further applied to modeling the thermal cracking of Venezuela petroleum vacuum residue [255]. Instead of using conventional kinetic modeling of the thermal cracking reactions of 400,000 vacuum residue molecules that needs an intractable solution of 1,000,000 material balance equations, the attribute PDFs, rather than molecules, were used, allowing them to simulate the pyrolysis reactor using only 2839 total material balance equations. The output attribute PDFs were then utilized to construct the product molecular composition and related properties [255]. The INGen software was used to create a comprehensive list of reactions for the three reaction families ascribed to the vacuum residue pyrolysis, which are cracking, aromatization, and coking. This detailed list of reactions is generated by looking for reactive moieties within each molecule in the system and performing the appropriate bond making and/or breaking. In INGen, chemical compounds are numerically assigned by their bond-electron matrices (BEMs), which are profound depictions of the atoms and connectivities (bonds) within molecules. The digital elements of the BEMs show the bond order between pairs of atoms within a given molecule. Bond formation and breaking are simply a matter of subtracting and adding the elements of the BEM for a given reactant that is represented by it. The reactive moieties are identified by INGen by means of looking for specific chains of atoms and bonds. With the suitable reaction operation, INGen identifies the product molecules and records the reaction. The reaction network, based on the three types (families) of reactions involved in the vacuum residue pyrolysis, cracking, aromatization, and coking, was established using the INGen software in about 30 s (Dell Precision T1500, processor: Intel (R) Core i7 870@2.93 GHz 2.93 GHz, memory: 4.00 GB). It consisted of 6274 reactions. The molecular-level kinetic model complexity was decreased to 21 adjustable parameters employing linear free energy relationships. The model showed a good agreement with experimental data irrespective of the diminished number of equations and adjustable parameters. It was also found that the accuracy of the SR molecular reconstruction models depends on the analytical data (distillation, paraffin–naphthene–aromatics (PNA) analysis, NMR of vacuum gas oils) [321]. The distillation curve data was identified as the main source of information about the variance of molecular attribute distributions (e.g., carbon number distribution), elemental analysis and PNA data help determine the mean values for various structure attributes, and 13C NMR data was strongly connected with information concerning the number of branches in molecules. Therefore, in the process of SR reconstruction of petroleum, diverse types of analytical data should be taken into account and it has to be determined which of them have a higher informative value, contributing to a more precise SR molecular model.

The SR method, that has been mainly used to construct the molecular composition of heavy oils, consists of four steps: parametrizing structural PDFs, sampling candidate molecules using Monte Carlo from a predefined library of attributes, calculating and averaging per-molecule properties, and iterating to convergence [43]. Irrespective of its molecular fidelity it faces the following challenges: (1) high dependence on the molecular library (absent substances cannot be reconstituted) [43]; (2) high computational cost generating 5000–50,000 molecules per iteration [376]; (3) stochastic output inconstancy because of random sampling and intractable likelihoods, which complicates convergence [14]. Some improvements were proposed such as ANN + GA hybrids to accelerate optimization [265], Markov chain Monte Carlo (MCMC) iterative sampling for greater flexibility [14,41], and Bayesian likelihood-free inference to regularize parameter estimation [14], all of which lead to ameliorated sustainability and efficiency of SR.

#### 2.1.2. Structure-Oriented Lumping and Hybrid Approaches Utilizing SOL Framework

**Figure 6:**
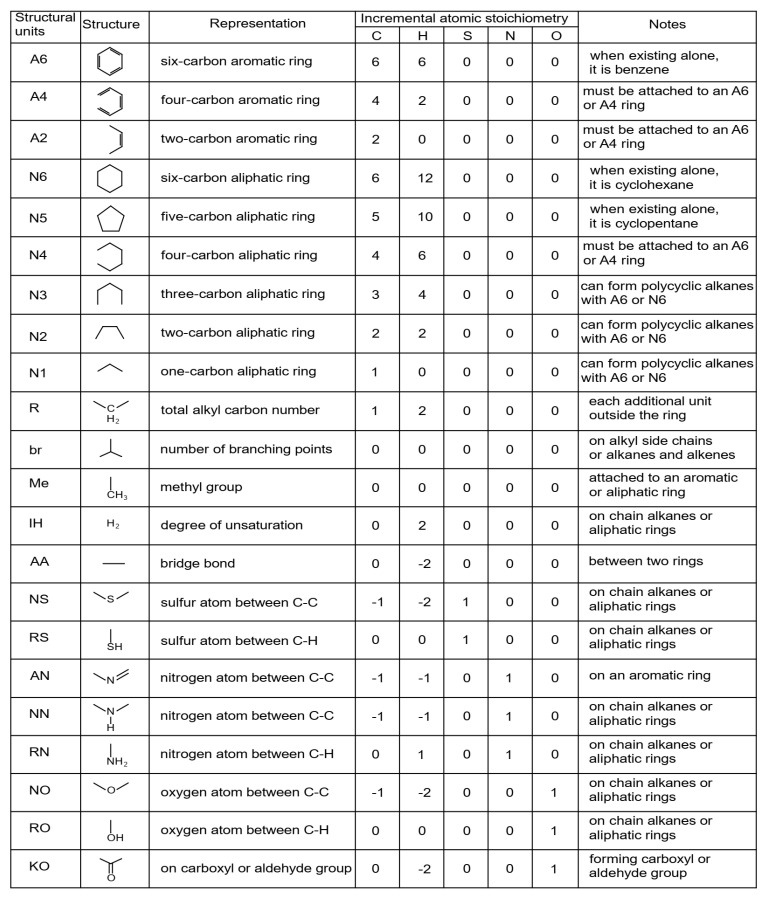
Structure-Oriented Lumping (SOL) method: 22 structural units and their meaning (modified from Quann and Jaffe [135]).

Quann and Jaffe [122] in 1992 introduced the structure vector concept to construct molecules of complex mixtures and their reaction, calling it structure-oriented lumping (SOL). The basic concept of SOL is that hydrocarbon molecules can be represented as a vector, with the elements of the vector depicting structural characteristics adequate to build any molecule. Every molecule in an oil blend is then portrayed by a structure vector. They defined 22 structural parameters, which form the structural vector of the oil molecule (see Figure 6). The atomic stoichiometry contribution of each element or structural group (parameter) of the vector is contained in the stoichiometric matrix. Structural groups have the stoichiometry to build a molecule gradually and are henceforth referred to as structural increments. SOL ensures a suitable architecture for handling chemical reactions as conversion of the feed structural increments to the product structural increments [135,147,150].

The original SOL method was primarily developed for gas oil and lighter fractions boiling below 593 °C wherein the oil composition can be represented by homologous series of molecules containing one aromatic ring (benzene, methyl–, ethyl–, propyl–, etc. benzenes). A combination of the SOL and group contribution methods to portray an oil fraction with 21 structural increments by the SOL method and to calculate molecular properties by the group contribution approach was proposed [70]. The method consisted of three steps: (1) SA algorithm optimization of structural distribution parameters (in this step 1000 molecules are obtained to match the experimentally determined elemental and SARA compositions of the heavy petroleum fraction); (2) genesis of a molecular library using Monte Carlo sampling of the optimized structural distribution parameters (20,000 molecules were created); (3) REM optimization of mole fractions of molecules. This method was applied to reconstitute two distinct VGOs. Twenty thousand molecules were generated for each VGO using the optimized parameters. For VGO-1 3460 unique molecules eventually remained in the molecular library to portray this particular VGO. For the second VGO 3411 unique molecules remained in the final molecular library. While the deviation of prediction of SARA composition was within the uncertainty of measurement, the simulated distillation showed under prediction of components evaporated below 10%, and between 70 and 90% of the compounds evaporated above 70%, that was outside of the uncertainty of the measurement. This suggests that either the correlations used to calculate the normal boiling point are characterized by a larger error or the simulated molecular distribution does not correspond very well to the real one.

A new heavy oil molecular reconstruction method based on SARA fraction simulated blending was found suitable for industrial applications and it enabled decreasing the reliance on molecular characterization data, while providing the reliability of reconstruction results [81]. It avails a non-linear least squares optimization algorithm, which can effectively improve the multi-solution problem in the process of solving molecular composition, while utilizing the SOL framework with 24 structure vectors. This method consists of two steps: (1) SARA simulated blending process; (2) composition adjustment based on bulk properties. SARA composition and the distillation curve of heavy oil are used as entrance variables in the first step, where the minimum of deviation between the distillation curve of the SARA mixture and the distillation curve of heavy oil is searched for by a non-linear optimization method. Assuming that the mixed distillation curve of SARA fractions obeys linear weighting and the weighting coefficients are the contents of the SARA fractions, this ensures that the optimization process can lead to a single solution. The molecular composition derived in the first step represents the input variables for the second step, wherein the minimum between measured and calculated bulk properties is looked for. This step allows corrections of the deviations entailed by the classification of the SARA phase diagram [440,441]. The first step avails a non-linear least squares optimization algorithm to minimize the deviation of the calculated from measured simulated distillation characteristics, optimizing the three parameters α, β and γ of the gamma PDF of the four SARA fractions (a total of 12 parameters). The molecular composition from the first step is calculated from the probability density function of SARA fractions with the optimized 12 parameters. The second step employs, instead of a non-linear least squares optimization algorithm, a genetic algorithm, because as a global optimization method, it has significant advantages over the least squares method in handling multi-variable complex problems. Using the composition data from the first step, the optimal molecular composition of the second step, that satisfies the minimum deviation between measured and calculated bulk residue properties, can be acquired in about 60 s by using the genetic algorithm. Six thousand nine hundred molecules were found adequate to reconstitute the chemical composition of the nine studied residual oils. A comparison between calculated molecular reconstruction, SARA composition, bulk properties and measured ones for nine heavy oils (four atmospheric residues and five vacuum residues), and the molecular composition of two atmospheric residues measured by HRMS showed a very high agreement. This suggests that the proposed method is capable of adequate molecular reconstruction of residual oils without necessitating molecular characterization data [81].

The SOL framework was also employed to reconstruct the molecular composition of the whole crude oil. The structure of each molecule constituting the whole crude oil was described by 21 structural increments. A two-step approach, consisting of constructing representative core structures as the first step and adding different lengths of side chains to each core structure to complete all the molecules as the second consecutive step, was availed [44]. Physical properties of reconstructed molecules were calculated by the group contribution method. A two-layer homologous series distribution was utilized to describe the distribution of the molecular library. In the first layer 19 types of core structures with five core structures in each type whose distribution was represented by an exponential distribution for the types of core structures and histogram distribution for the core structures were used. In the second layer side chains with a length of 1–50 carbons are added to each core structure, and the gamma distribution is availed to describe the carbon number distribution of the side chains. Based on the two-layer homologous series distribution and probability density function, the mole fractions of all molecules were computed. The physical properties of the reconstructed crude oil were calculated using the mole fractions of all molecules, their calculated physical properties and the mixing rule. The particle swarm optimization (PSO) algorithm was availed to minimize the deviation between measured and calculated crude oil properties. Fifty-five crude oil datasets were availed in the crude oil reconstitution process in the study of Zhang et al. [44], while in another study of Zhang et al. [42] 42 crude oil sets were utilized.

SOL was applied to build a new-generation FCC process model in 1999 [150], that availed more than 3000 FCC feed molecules using over 60 reaction rules to create a complex network of upward of 30,000 elementary chemical reactions. The FCC SOL model was found to correctly predict yields, product composition, and quality for a wide range of operating conditions and feedstock quality. The original SOL method was further expanded in 2005 by inclusion of additional structure increments, which contain more than one ring aromatic structure and nickel and vanadium porphyrins, increasing the initial 22 structural parameters to 24 in order to model petroleum residues [103]. A set of initial residue molecules, coined as “seeds”, was proposed, enabling chemical conversions to obtain products whose properties and compositions cover the diversity of properties and compositions of the different residue refining processes. Nine types of molecular structures, each with 7–9 nuclei, were used to create 56 seeds, and applying the reaction rules of petroleum chemistry to all these seed molecules resulted in the generation of 245 molecules, 44 of which had a single nucleus [103].

The next step in modeling the FCC process at the molecular level is to avail a non-linear least squares method to calculate the amount of saturated hydrocarbon (Sa), aromatic (Ar) hydrocarbon, resin–asphaltene and elemental content (C, H, S, N) in the FCC feedstock utilizing the SOL framework. Here, 18 structural vectors and 38 kinds of homologous series such as alkanes, alkenes, cycloalkanes, aromatic hydrocarbons, polycyclic aromatic hydrocarbon, etc. were chosen to build 391 “seed” molecules by adding –CH_2_– side chains to the homologous series. The 391 seed molecules consisted of 62 kinds of paraffin, 11 kinds of olefins, 94 kinds of naphthenic hydrocarbons, 187 kinds of aromatic hydrocarbons, and 37 kinds of condensed ring hydrocarbons. Sixty-six reaction rules were proposed and 100 steps of circular reactions were assumed to model the whole reaction network [228]. The entire reaction network consisted of 162,787 reactions and the products included 1078 types of molecules. The average relative deviation in prediction of molecularly reconstituted VGO feed properties, C, H, S, N elemental content, saturates, aromatics, resin content, and density, was 2.6%, while that of the FCC yields was 4.1%. The incorporation of the non-linear least squares method in the SOL framework enabled improvement in the accuracy of prediction of the FCC VGO characteristics.

The SOL method was further exploited to model the FCC of feedstock containing residue, a mixture of hydrotreated residue oil (70%) and atmospheric gas oil (30%) (boiling between 330 and 700 °C) [267]. The conventional 22 structural increments were availed to reconstruct the residue containing FCC feed. Ninety-two seed molecules were selected based on the results of positive-mode APPI FT-ICR mass spectrometry. A molecular library of 14,692 molecules was created using the molecular weight distribution of the FCC feedstock and addition of 0–50 –CH_2_– side chains to the seed molecules. The relative content of these 14,692 molecules in the FCC feedstock was determined using the SA algorithm. Ninety-five reaction rules were chosen to represent the entire reaction network. The CPU processing needed to perform the simulation of reaction network took 25–30 h (computer configuration: processor, Intel(R) Xeon(R) CPU E5-26430 at 3.30 GHz (dual processor); internal memory, 32.0 GB; system type, 64-bit operating system). The reaction network of the FCC process was represented by 702,943 reactions. The relative average deviation of calculated FCC yields from experimental ones was 5.9%. This simulation of the residue FCC process at the molecular level allows for prediction of product distribution along the riser reactor and understanding of the mechanism by which different operating conditions can affect both yield and product quality.

The new FCC technology—maximizing isoparaffins (MIPs) while processing a mixture of atmospheric residue and vacuum waxy oil—was also modeled at the molecular level by exploiting the SOL framework with a data model derived with the CatBoost algorithm [329]. The conventional SOL mechanism model, which can have over 14,000 molecules and 700,000 reactions, was reduced to a more manageable set of 3078 molecules, 5216 reactions while the accuracy of yield prediction was not sacrificed due to the implementation of the CatBoost algorithm, resulting in a significant reduction of computing time reaching as low as 1 min. The average absolute deviation of predicted yields from the measured ones was 0.5%, while that of the PIONA composition of obtained gasoline was 1%.

The combined action between SOL using the conventional 22 structural increments (see Table 2), together with a neural network algorithm, allowed the construction of an FCC SOL-ANN property prediction model [385]. The SOL-ANN hybrid model of gas oil catalytic cracking leverages the detailed structural information inherent to SOL to predict key fuel properties like research octane number (RON) and cetane number (CN) directly from the simulated molecular composition of the products. The simulated boiling point, density, RON, motor octane number (MON), and CN of molecules were predicted accurately and reliably through the SOL-ANN property prediction model and the fitting correlation coefficients R^2^ of the boiling point, density, RON, MON, and CN were 0.991, 0.993, 0.983, 0.978, and 0.989, respectively. This research demonstrates a practical application of the hybrid model. By increasing the reaction temperature from 490 °C to 510 °C in the FCC process simulation, specific changes in molecular composition (increased aromatics and olefins) and, crucially, the resulting fuel properties: a 2.96-unit increase in gasoline RON and a 1.37-unit decrease in diesel CN, were predicted. This approach effectively bridges the gap between highly detailed but computationally intensive mechanistic models and the need for rapid, accurate predictions for process optimization, demonstrating a clear path forward for molecular-level process simulation.

The SOL approach was found appropriate to investigate the behavior of sulfides boiling in the range of naphtha and diesel during FCC of heavy oil. The reaction network built using the SOL framework consisted of 110,000 reactions, allowing for tracking the specific generation and conversion pathways of individual molecules, such as different sulfide classes (mercaptans, thioethers, thiophenes, etc.) [337]. The employed SOL approach availed 24 structural increments to build a matrix of 4148 structural vectors representing the feed oil. This balances molecular detail with computational feasibility, avoiding the need to model every single compound individually. The research exemplifies a trend towards modeling not just hydrocarbons but also non-hydrocarbon species (sulfides) at a molecular level. This is crucial for addressing modern refining challenges like deep desulfurization to meet stringent fuel standards. The model allows for quantitative investigation of how operating conditions affect the sulfide content in different product fractions (gasoline and diesel).

The SOL framework was also employed for the development of an integrated molecular-level and mixed-fraction model for the direct catalytic cracking of whole crude oil [336]. Instead of using a purely molecular-level model (which can be computationally expensive) or a purely lumped model (which lacks mechanistic detail), Zhou et al. [336] combined both. This approach balances mechanistic detail with computational practicality through adequate simplification. The mixed fraction structure component represents a pragmatic simplification. It suggests that the model may group some complex heavy fractions (which are difficult to characterize molecule by molecule) into broader lumps, while modeling the cracking of lighter, more defined fractions at a detailed molecular level. This research is a perfect example of the adequate simplification rules, making the complex problem of simulating whole crude oil cracking more tractable. Zhou et al. [418] introduced a novel optimization framework, SOL-PINN-BO, to vanquish the elaborate multi-objective optimization challenges of crude oil direct catalytic cracking (UPC) technology. The SOL model allows accurate forecasting of functional group compositions in naphtha and diesel products at the molecular level. By leveraging PINNs, the development of molecular-level kinetic models is speeded up, and crucial reaction mechanisms controlling olefin formation during the reaction process are more easily identified. Eventually, this effective SOL–PINN substituent model is coupled with Bayesian optimization to enable multi-objective process optimization. The catalytic cracking of Daqing crude oil was explored in the research of Zhou et al. [418]. Due to its low heteroatom content the 24 characteristic structural units employed in the extended SOL method were decreased to 22. The characterization process of the Daqing crude oil involved measurement of bulk physical properties along with molecular composition analysis by comprehensive two-dimensional gas chromatography coupled with time-of-flight mass spectrometry (GC × GC-TOFMS). It allowed construction of a representative molecular library containing over 50 core molecules. The addition of –CH_2_- side chains to these 50 core molecules gave a comprehensive Daqing crude oil molecular database including >2000 structural units. Molecular properties were calculated using group contribution methods [61]. Molecular properties were computed availing group contribution methods [61] with composition distributions optimized via simulated annealing algorithms. Validation confirmed excellent agreement between optimized database properties/composition distributions and experimental measurements, with all relative errors below established thresholds. Employing SOL methodology, a catalytic cracking network with 4320 reaction pathways was built. The kinetic parameters of this reaction network were calibrated against pilot-scale experiments (error < 5%), precisely characterizing reaction mechanisms and product distributions. A PINN was established to replace the SOL model, embedding mass conservation and reaction kinetics into its loss function. The SOL-PINN accepts operating parameters as inputs and predicts product yields and emissions, maintaining molecular-level accuracy with a 12-fold computational speedup. The SOL-PINN substitute was coupled with Bayesian optimization. Bayesian optimization smartly investigates intricate operating parameter spaces (e.g., reaction temperature, catalyst-to-oil ratio, space velocity), availing Gaussian process substitutes and advanced acquisition functions like expected hyper-volume improvement (EHVI). Examined by industrial cases, this framework indicates 600-times quicker optimization than conventional methods with <3% key indicator prediction error.

The SOL framework was additionally employed to investigate the secondary reactions of gasoline, which takes place in the FCC process in severe operating conditions (reactor temperature of 600 °C and catalyst-to-oil ratio from 13.2 to 13.9 wt./wt.) [194]. The SOL method was combined with Monte Carlo to model the process at the molecular level. Nineteen SOL increments were used by the Monte Carlo method to construct more than 4000 molecules for the three studied FCC gasoline samples. The simulation results confirmed that, the larger the number of representative molecules, the higher the accuracy of predicting gasoline properties and the longer the simulation time. It was concluded that the optimal balance between accuracy of property prediction and CPU time was achieved when the number of representative molecules did not exceed 5000. The reaction network, modeled by 60 reaction rules of normal and isoparaffins, normal and iso-olefins, naphtenes, and aromatics, was divided into 400 small processes, implying that the feed conversion is simulated by the product matrix, which underwent a 400-step process. The average relative deviation of predicted yields from experimental data of dry gas, LPG, gasoline, LCO, HCO and coke was 2.96%, while that of the hydrocarbon composition was 4.8%. The proposed new combined SOL-Monte Carlo method demonstrated that molecular modeling can accurately predict both yields and product quality. Pan et al. [242] employed the same data for catalytic cracking of three FCC gasoline samples from the research of Yang et al. [194], aiming to improve the simulation process of the secondary reactions of FCC gasoline at the molecular level by introduction of a novel combined method that ally the SOL, Monte Carlo and REM approaches. In contrast to the study of Yang et al. [194], who set a limit of maximum 5000 molecules to reconstruct the FCC gasoline, Pan et al. [242] represented the same FCC gasoline samples by 30,000 virtual molecules. The new combined method featured much shorter time to reconstruct the FCC gasoline (0.8 h) than the traditional Monte Carlo method (between 3 and 14 h) when using Intel Core I5-2520M CPU. The use of the new method provided improvement in the accuracy of FCC gasoline property prediction by 24% compared with the combined SOL and Monte Carlo method utilized in the research of Yang et al. [194].

The SOL approach was also utilized to create coupling models that connect different refinery units (in this case FCC and hydrotreating) [365]. This represents an evolution from single-unit molecular models to system-level simulations. The key advancements are: (1) the use of a detailed molecular matrix (using SOL) to enable tracking of environmentally regulated compounds (sulfur, PAHs) at a mechanistic level; (2) the development of a fractionation tower simulation system to achieve accurate material flow coupling between processes, which is a critical step for realistic plant-wide optimization; (3) a clear focus on applying the model to solve immediate industrial challenges, such as meeting clean fuel specifications. This research demonstrates that the challenge is no longer just building a molecular model for one process but effectively integrating these models to reflect the interconnected reality of a refinery. The FCC–diesel hydrotreater coupling model was utilized to evaluate the dependence of the distributions of hydrocarbon compositions in diesel in the DH process on the FCC reaction conditions (reaction temperature variation between 500 and 560 °C) and on the diesel hydrotreater operating conditions (reaction temperature variation between 300 and 360 °C). The research in this field continued with the development of coupling models by building of a molecular-level coupling model of FCC and an FCC gasoline hydrotreater (FCCGH) availing the SOL framework. The established model contained 96 FCC reaction rules and 24 FCCGH reaction rules and the reaction network comprised approximately 120,000 reactions [366]. Using the developed model it was found that the content of benzene in heavy gasoline at the outlet of the FCCGH process decreased from 1.01 wt.% to 0.66 wt.% while cyclohexane content was augmented from 0.30 to 0.35 wt.%. when the FCC reaction temperature was increased from 500 to 560 °C.

The SOL method was also applied to reconstruct the feedstock for steam cracking [211]. In this research the number of structural increments to model naphtha fractions was seven in contrast to 19 in the previous study reconstructing FCC naphtha [194] that had similar molecular weight and boiling range. The naphtha steam cracking feedstock was represented by 132 seed molecules, and 74 reaction rules were applied to model the entire reaction network. The behavior of these 132 seed molecules during steam cracking in various operating conditions was described availing Materials Studio to compute reaction rate constants and the Runge–Kutta method to solve simultaneous differential equations. The relative average deviation of the predicted methane, ethane and butadiene yields from the experimental ones is 6.2%, which is twice as high as that of Yang et al. [194]. The studies utilizing the SOL framework to reconstruct naphtha fractions and their transformation during FCC [194] and steam cracking [211] suggest that, the higher the number of structural increments, the higher the precision of yield prediction, although the number of reaction rules varies.

The SOL method was also employed to reconstruct two residue feedstocks from Sinopec for delayed coking running at 480 °C [230]. Here, 22 structural increments were availed to construct 92 single-core seed molecules and 46 multi-core seed molecules. After addition of –CH_2_– to the seed molecules 7004 molecular lumps were obtained. Ninety-two reaction rules were introduced to describe the reaction network of the delayed coking process. The relative average deviation of residue feedstock properties predicted from the molecular composition was 12.7%, with the highest deviation for the composition of elements sulfur (34.4%), oxygen (45.9%), nitrogen (19%), vanadium (23.5%), and Ni (18.6%), suggesting that the molecular reconstitution was not very accurate either because of the analytical data used or the algorithm employed. However, the prediction of yields was characterized by a much lower average relative deviation than the residue properties—2.5%—supposing that the inaccurate prediction of elements mentioned above is not crucial for the delayed coking modeling. The same SOL method was further examined to model the delayed coking of four different residues: Saudi: Diaz: Soros: Oman (2.6:2:0.2:5.2); Iran: Soros: Saudi: Diaz (0.5:1:6.5:2); Basra: Kuwait: Mundo (3.6:2.4:4); and Soros: Kuwait: Djeno (1.5:5.3:3.2), that was running in variable operating conditions (e.g., reaction temperature variation between 480 and 500 °C) [225]. It was assumed that the four examined residues could all be reconstituted using 7004 molecular lumps. This is analogous to the admission of Law et al. [300] that all petroleum oils are constructed by the same number molecules but in different proportions. It was admitted that the diverse properties of the four investigated residues were a result of the different relative contents of these 7004 molecular lumps. Therefore, if the relative content of molecular lumps was known, the molecular composition matrix of the feedstock could be constructed after representing each molecular lump with the 22 structural vectors. A simulated annealing algorithm (SA) was availed to simulate the feedstock molecular composition. The average relative deviation of predicted yields from experimental ones was found to be 2.9%. This deviation is a little bit higher than that reported for the two residues from Sinopec and it may be a result of the different characteristics of the residues being cracked in the delayed coking process.

The SOL framework was further exploited for the development of a molecular-level reaction kinetic model of delayed coking process with a reaction network consisting of 81,081 reactions to track the reaction pathways of sulfur compounds [325]. Here, 24 structural increments were availed to build the petroleum residue feed molecular matrix comprising 2944 structural vectors based on the residue characteristics: the contents of SARA fractions, the contents of carbon residue, the number of aromatic rings, the number of naphthenic rings, and the element contents. The calculated properties, such as those mentioned before, and the distillation curve were in very good agreement with the measured ones (average relative absolute error of 1.6%, and maximum error of 6.9%). The molecular-level model allowed tracking the transformation of sulfur compounds and accurately predicting the distribution of sulfur compounds in coking gasoline, as well as revealing the influence of reaction conditions on the transformation of sulfur compounds in the delayed coking process at the molecular level.

The conversion pathways of polynuclear aromatic hydrocarbons and heteroatomic compounds during gas oil hydrocracking were tracked utilizing the SOL modeling framework [370]. The conventional 22 structural increments of the SOL method (see Table 2) were used to describe gas oil feed molecules and their transformation was followed through the hydrocracking process. The reconstitution process of the feed generated a molecular composition matrix that consisted of 3045 structural vectors. About 89,000 reactions were generated to represent the hydrocracking reaction network. The molecular-level hydrocracking model very well described the transformation of normal and isoparaffins, naphthenes and aromatics with carbon numbers between C_4_ and C_21_ (boiling in the range of light and heavy naphtha, kerosene and diesel), showing its high reliability.

A molecular-level reaction kinetics model of a heavy oil blend that consisted of vacuum residue, vacuum gas oil, FCC heavy cycle and slurry oils (FCC HCO and FCC SLO) in a hydrotreating process based on the SOL framework with a reaction network involving 24,422 cracking reactions, 1227 ring opening reactions and 2494 hydrogenation saturation reactions was developed [408]. A complex analytical procedure comprising sSFEF, column chromatography separation (CCS), simulated distillation, elemental composition, and molecular composition determined by FT-ICR MS was availed. Based on these analytical procedures, it was found that the specific structure and content of each molecule in the feed residual oil were very hard to accurately assign. Nevertheless, 241 seed molecules were initially determined according to the composition information of residual oil feed. The addition of side chains to the 241 seed molecules using the simulated distillation curve as a constraint resulted in a matrix containing 7902 structural vectors. A simulated annealing algorithm was applied to obtain the content of the 7902 structural vectors in the matrix residue feed. The average relative absolute error in the calculated hydrocarbon group compositions, element and carbon residue and the industrial data was less than 3.0%. The model reveals the transformation pathways of hydrocarbon and non-hydrocarbon molecules in the residual oil hydrotreatment process and is capable of predicting the effects of reaction conditions on product distribution at the molecular and group composition levels.

The hydrotreatment of FCC gasoline was also modeled at the molecular level by employing an extended SOL framework that availed 40 structural increments [409]. The reaction network constructed consisted of 4037 reactions. The analytical technique employed in this study was two-dimensional gas chromatography with time-of-flight mass spectrometry (GC × GC-TOF MS) because of the higher separation and detection efficiency compared to the conventional gas chromatography with flame ionization detector (GC-FID). The model allows tracking the transformation of the molecules of hydrocarbons, sulfides and nitrides in the reactions of their formation and consumption. It also enables the evaluation of the effects of reaction conditions on the composition and octane number of hydrotreated gasoline.

A molecular-level kinetic model of hydrotreated shale oil based on the SOL framework was also established [413]. The hydrotreated shale oil is a complex organic mixture boiling in the range 100–700 °C that is very difficult to analyze without preliminary fractionation to naphtha, diesel, VGO, and vacuum residue. The characterization techniques used for the different shale oil cuts were as follows: naphtha—paraffins, isoparaffins, olefins, naphthenes, and aromatics (PIONA) GC analysis, diesel and VGO − GC × GC-TOF MS and VR − ^1^H-NMR. Twenty-two structural units were availed to build the hydrotreated shale oil molecular matrix (except Ni, V). The information obtained from the group composition and structure characteristics of each shale oil cut was used for the selection of a total of 56 core molecules to represent homologue molecules in shale oil. Adding side chains to the 56 core molecules resulted in the construction of a shale oil matrix composed of 1691 structural vectors. The group contribution method was employed to calculate molecular properties. A simulated annealing algorithm was availed to optimize molecular composition, providing an average relative absolute deviation between calculated and measured properties of 1.65%. The developed molecular-level kinetic model can calculate hydrocarbon distribution in the reaction process and create correlation between reaction conditions and product distribution. Based on the molecular-level kinetic model, process simulation software including reaction condition optimization and process deep analysis functions was developed.

A kinetic model for a naphtha catalytic reformer at the molecular level by applying a modified SOL method to fit the catalytic reforming and a structural increment to discern between the xylene isomers was also developed. To describe the chemistry of the naphtha reforming process 140 molecules were involved and 697 reactions were generated following 16 reaction rules. After that a mathematical model was established, that included a reaction kinetic model and a reformer reactor model. The use of the linear free energy relation (LFER) method to reduce the number of kinetic parameters is a crucial adequate simplification rule. Instead of needing to determine a unique kinetic parameter for every single reaction, LFER allows parameters to be estimated based on a smaller set of representative reactions and thermodynamic properties, making the model more robust and faster to develop. The novelty in this study is the introduction of specific structural increments to differentiate between critical isomers (xylenes), enhancing predictive accuracy for valuable products.

The SOL framework was found flexible enough to be capable of modeling both light and heavy oils and residues, balancing molecular fidelity with computational tractability.

#### 2.1.3. Reconstruction by Molecular Type Homologous Series

**Figure 7:**
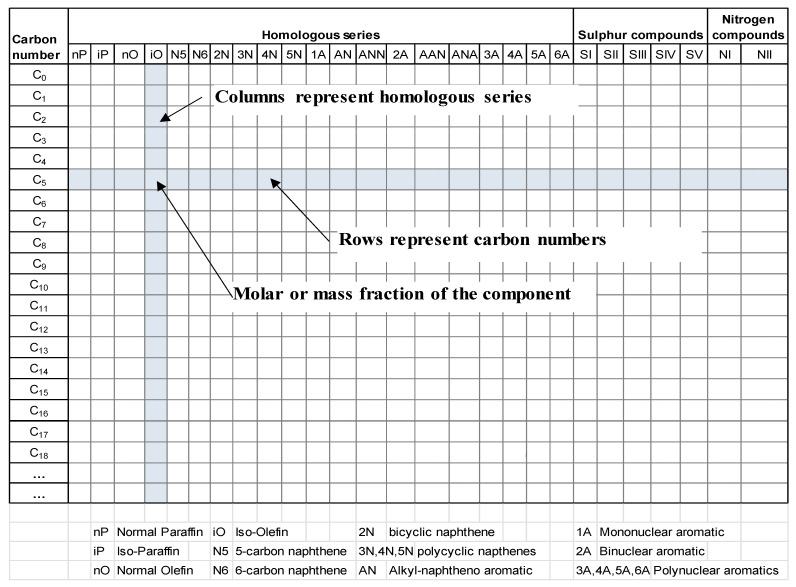
Molecular Type Homologous Series (MTHS) matrix representation (modified from Peng [151]).

Peng [151] proposed a new framework to model molecular composition of petroleum fractions where the oil mixture is represented as a matrix in which the rows represent carbon number (molecular size) and the columns depict the homologous series (Figure 7). It was coined molecular type homologous series (MTHS). The number of homologous series was 22, similar to the number of structural groups of SOL [58]. Peng’s MTHS molecular matrix representation framework was applied by Hou et al. [164] to optimize the performance of naphtha hydrotreating, catalytic reforming, stabilizing and blending of gasoline. They showed that the MTHS matrix could be expanded from the original 22 to 30 homologous series and from the original 22 carbon numbers to 45. To improve the precision and practicality of the MTHS matrix concept, Aye and Zhang [58] created a new procedure to convert the physical properties of a hydrocarbon mixture into molecular data in the compositional matrix and inversely. Gomez-Prado et al. [64] extended the applicability of the MTHS matrix concept from naphtha and diesel used previously to heavier petroleum fractions. They reshaped the original MTHS matrix by replacing the carbon numbers in the modified matrix (mMTHS) by the boiling point. In contrast to the previous study of Zhang [152], whose maximum boiling range of petroleum oils, being reconstructed by the MTHS matrix method, was that of diesel (approximately 350 °C), and that of Aye and Zhang [58], who employed direct physical properties, in the study of Gomez-Prado et al. [64] characterization parameters were availed. Gomez-Prado et al. borrowed the idea of the better separation ranges between the different homologous hydrocarbon groups of the characterization factors, established by Riazi and Daubert [442]. The characterization approach proposed by Gomez-Prado et al. [64], which uses mMTHS, has been shown to be capable of reconstructing petroleum oils ranging from naphtha to residues (boiling up to 700 °C), by calculating the fraction of each component through minimizing the discrepancies between the bulk and calculated characterization parameters. The characterization approach proposed by Gomez-Prado et al. [64] that avails the mMTHS was reported to be capable of reconstructing petroleum oils ranging from naphthas to residues (boiling up to 700 °C) by calculation of the fraction of each component by minimizing the differences between the bulk and the calculated characterization factors. Ahmad et al. [217] further amended the MTHS matrix molecular reconstruction method, in which the properties of petroleum fractions were calculated based on the properties of individual components, rather than using the characterization parameters as proposed by Gomez-Prado et al. [64].

Ahmad et al. [217] compute the physical and thermodynamic properties of individual components of petroleum fractions by applying group contribution methods, while Gomez-Prado et al. [64] utilize molecular structure–property correlations developed by Zhang [152]. The method of Ahmad et al. [217] for matrix generation provides the molecular composition of petroleum oils without the need for detailed chemical analysis. A graphical representation of the computing procedure of the MTHS matrix method is given in Figure 5. An extension of the applicability of the MTHS framework from molecular reconstitution of light to medium petroleum fractions [48] to heavy oils was proposed by Song et al. [41]. This expansion can be done by (1) development of an elaborate DBE-based property database, that defines 23 hydrocarbon classes including the whole crude oil boiling range from light naphtha to residue compounds boiling above 1000 °C; (2) inclusion of extra columns in the MHTS matrix for heavy polycyclic aromatics and for distinct heteroatom-containing subclasses allowing explicit modeling of sulfur- and nitrogen-rich species with structural significance; (3) insertion of conservation of carbon, hydrogen, sulfur, and nitrogen into the objective function providing atomic-level consistency. Song et al. [41] tested their amended MTHS procedure to reconstitute the molecular composition of hydrotreated atmospheric residue feedstock, for residue FCC slurry, FCC light cycle oil and FCC gasoline. The reported results are promising for this novel MTHS platform, indicating a very good agreement between measured and calculated properties of tested light, middle and heavy oils, suggesting that it could be used for further process simulation and optimization purposes. Moreover, the molecular reconstitution process takes only between 3 and 36 s (laptop equipped with an AMD Ryzen 9 5900HS processor (8 cores, base clock 3.3 GHz and 16 GB RAM using CasADi and IPOPT)).

The MTHS framework was also applied for molecular reconstitution of whole crude oil based on a predefined set of molecular models consisting of representative molecules for light and medium fractions and average molecules for heavy fractions [9]. The forecasting of molecular composition was obtained by fitting the true boiling point (TBP) curves to the API gravity data, providing agreement with the SARA fraction contents in the investigated crude oils. Here, 151 molecules were classified as saturates (78 molecules), aromatics (45 molecules), resins (16 molecules) and asphaltenes (12 molecules) whose boiling points were calculated using the group contribution method [443], while the densities of molecules were computed employing Material Studio [9]. The computed results showed a good agreement with the SARA fraction content, density and boiling points of 28 studied crude oils. Aspen Plus software process simulator was used to test the correctness of the molecular content distribution of crude oil in the simulation of the distillation process. The simulated results indicated that, while some fraction properties were very well predicted, others showed a noticeable deviation between simulated and actual values, suggesting that more accurate molecular structures should be introduced in the model. In addition, incorporating more properties of studied crude oils as constraints or optimization objectives to increase the accuracy of the model in predicting the molecular content distribution of crude oil is needed.

MTHS was originally developed for light and medium petroleum fractions. The MTHS matrix is characterized by a two-dimensional grid: carbon number or boiling range as rows, molecular type as columns. Unlike the SOL framework, MTHS is able to distinguish between isomers, thus reducing the number of unknown variables, that enables simplifying the model. The original MTHS has two main limitations: (1) it is not suitable for heavy fractions due to the difficulty in building molecular libraries; (2) it is strongly dependent on high-resolution analytical data, which limits its use to well-characterized fractions. However, recent work by Song et al. [41] proposed a recapitulative database-integrated MTHS framework that allows for reliable characterization of both light and heavy petroleum fractions. Its high prediction accuracy and computational efficiency offer a reliable basis for advanced reaction modeling, process simulation, and optimization. Future applications of the database-integrated MTHS framework are expected to be focused on its deep integration with chemical process simulation and optimization systems. A key direction is the implementation of MTHS as a unified molecular input for reaction models (e.g., FCC, hydrocracking) of mixed feedstocks. At the same time, MTHS is expected to characterize product streams to predict online properties such as density and viscosity, tracking their changes depending on operating conditions.

#### 2.1.4. Reconstruction by Entropy Maximization and Hybrid Methods Utilizing REM Approach

**Figure 8:**
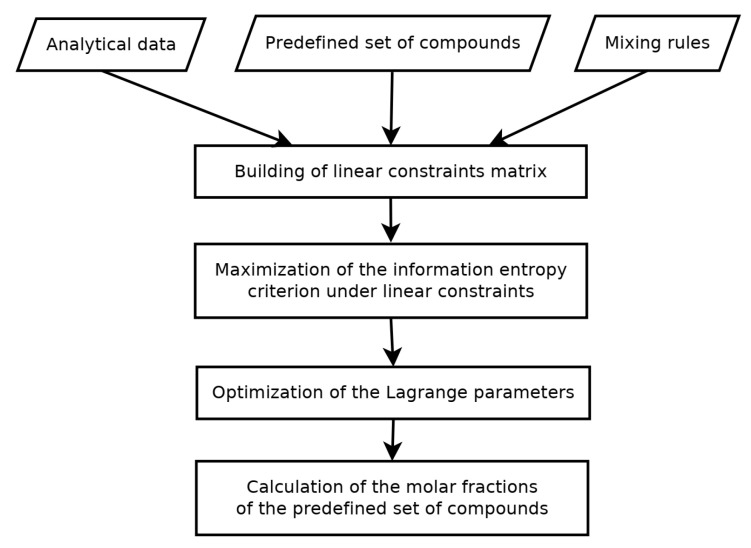
REM petroleum oil reconstitution process diagram (modified from Feng et al. [67]).

Hudebine et al. [169] offered a molecular reconstitution technique, which applies the maximizing information entropy principle, coined “reconstruction by entropy maximization” (REM). It applies the Shannon entropy, adopted from Shannon’s information theory, which is a measure of the homogeneity of the probability distribution [62]. Higher values of Shannon entropy imply more uniform distribution of the parameters studied. When petroleum oil is reconstructed the probabilities are replaced by the molar fractions of the various molecules present in the oil blend. A graphical representation of the REM is depicted in Figure 8. REM consists of three steps: (1) generation of a representative molecular database of the oil mixture to be reconstructed (for light oils such as naphtha, the molecular database can be created by using gas chromatographic techniques, while for heavier oils the molecular database can be created by another reconstruction method, such as the stochastic reconstruction, for example); (2) defining the constraints related to the distinct petroleum analyses for the oil blend; (3) tuning the molar fractions by maximizing the information entropy criterion considering the constraints defined in step 2.

The REM method was tested along with artificial neural networks (ANNs) and multiple linear regression (MLR) for reconstruction of the molecular composition of three naphtha samples as detailed naphtha hydrocarbon composition can be measured in a reasonable time [209]. It was found that ANNs and MLR can precisely reconstitute the molecular composition of naphtha in the case that the reconstructed naphtha has similar properties to those of the large number of training naphthas used to develop these reconstruction methods. The ANN model was determined to be superior to the MLR. The ANN model outperformed the REM method in accuracy when the naphtha characteristics were within the application range defined by the training set. Outside this range, however, the accuracy of the ANN method was significantly reduced, while that of REM was not affected by the characteristics of the naphtha considered.

REM was allied with the stochastic reconstitution approach introducing a two-stage stochastic reconstruction–entropy maximization (SR-EM) hybrid model aiming to overcome the shortcomings of each of these methods: computational effort, objective function noise, and sensitivity to the initial set of molecules [174]. For example, during reconstitution of LCO by SR underestimation of monocycloparaffins and overestimation of indanes/tetralins were noted [174]. This deviation of the simulated hydrocarbon content from that measured by mass spectrometry was ascribed to the SR internal limitations. Furthermore, the SR method proved to be more time-consuming in contrast to the REM method [174]. REM, on the other hand, also has disadvantages, such as (1) strong dependence on the initial set of molecules; (2) high sensitivity to the predefined initial set of molecules and the properties of the mixture calculated on their basis, when they are too far from the analytically measured values. de Oliveira et al. [61] evolved the SR-REM method by developing a new indirect two-step reconstruction algorithm. The first step—SR—was used to construct a single reference equimolar set of molecules, while the second step—REM—completed the molecular reconstruction of diverse petroleum fractions, enabling reduction of computational load and representing various vacuum residues from different origins with the same set of molecules. This novel SR-REM method is suitable for molecular reconstruction of a great number of vacuum residues because the SR step, that requires about a day, is followed by the REM step, that needs only a few seconds of CPU time. In the SR step a set of 20,000 molecules was generated, that was proved sufficient to successfully reconstitute the chemical composition of eight distinct vacuum residues (Iraq (Buzurgan), Russia (Ural), Mexico (Maya), Canada (Athabasca), Indonesia (Ardjuna and Duri), Congo (Djeno), Saudi Arabia (Arabian Medium)). The results of de Oliveira et al. [61] suggest that both the number of molecules of 20,000 along with the new upgraded SR-REM method seem to solve the issue of unique molecular composition discussed in the work of Cambell and Klein [137]. de Oliveira et al. [104,105,106] applied the SR-REM methodology to simulate the molecular composition of Athabasca and Ural vacuum residues and then as a second step to simulate the effect of the hydroprocessing reactions on the generated set of molecules using a kinetic Monte Carlo (kMC) method, coined as Gillespie’s stochastic simulation algorithm (SSA). The hybrid SR-REM method was also availed to reconstruct 15 different VGOs [335]. The characterization data of the 15 VGOs obtained from measurements of density, elemental composition (C, H, S and N), simulated distillation, hydrocarbon type distribution and S-heterocycles, analyzed by two-dimensional gas chromatography with a flame ionization detector and sulfur chemiluminescence detector, respectively, was used to reconstruct their molecular composition. It was found that the generally availed properties such as density, elemental composition, PNA distribution and boiling point distribution were not adequate to generate an appropriate molecule library for VGO. Thorough compositions of naphthenes and aromatics related to ring number as well as S-heterocycles were also needed. The general molecule library obtained by the SR method for the VGO consisted of 3023 unique molecules with a gamma distribution within the carbon number range of 9–45. With this general molecule library all 15 diverse VGOs were reconstructed by the REM method. While in the study by de Oliveira et al. [61] a 20,000-molecule library was found sufficient to reconstruct the molecular composition of various vacuum residues, the research of Wang et al. [335] showed that the VGO library could be seven times smaller, most likely because the composition of VGO is less complex than that of the VR. The average absolute relative errors in predicting measured VGO properties were reported to be less than 10%.

REM has limited application as a stand-alone molecular modeling framework and can be applied to petroleum fractions in the naphtha and diesel ranges. However, for heavy oils such as VGO and vacuum residues, it can be applied in combination with the SR approach.

#### 2.1.5. Reconstruction by Structure Unit–Bond-Electron Matrix (SU-BEM)

**Figure 9:**
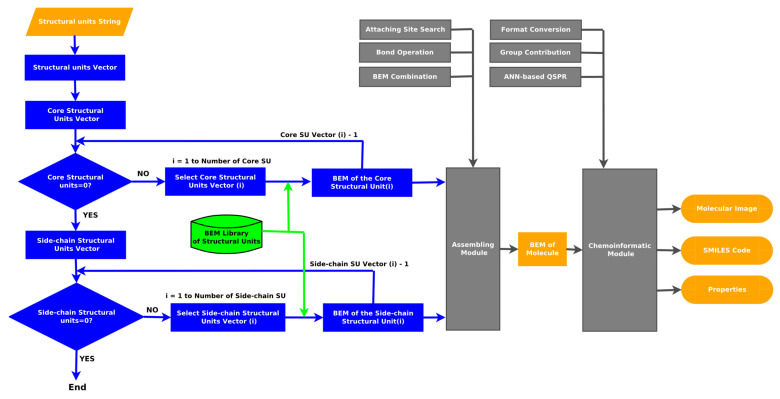
Principle of the operation of SU-BEM method (Modified from Feng et al. [67]).

Feng et al. [67] proposed a novel methodology for representing and reconstructing the molecular composition of petroleum fractions, coined as SU-BEM (Figure 9). It is a foundational piece of research that introduces a new conceptual framework, which can then be applied using various analytical inputs. The SU-BEM is in its essence a hybrid framework that borrows from the SOL method’s “structural vectors” to represent molecules based on core building blocks and the BEM concept pioneered by Klein’s group [187] that describes molecules based on atom type and connectivity. The SU-BEM framework aims to be both mechanistically detailed (like BEM) and computationally tractable (like SOL). The SU provides a simplified representation for speed, while the BEM ensures the fundamental chemical information is not lost. Feng et al. [67] applied their new molecular modeling technique SU-BEM to reconstruct the molecular chemical composition of diesel, VGO, and FCC slurry oil (SLO). While the diesel properties were well predicted, the density of VGO and that of FCC SLO were underpredicted, while the hydrogen content of FCC SLO was overpredicted, suggesting that the analytical methods used have not provided the required information as an input for the SU-BEM framework. Chen et al. [68] developed a SU-BEM molecular model for the FCC of heavy oil. Their approach addresses the computational challenge by representing molecules with simplified structural units that retain critical atom-connectivity information. The method successfully generated a complex reaction network (~3800 product molecules from ~7500 reactions) and, after parameter estimation, successfully predicted product yields, demonstrating a successful trade-off between mechanistic detail and computational efficiency, paving the way for more precise optimization of refining processes for heavy feedstocks. Chen et al. [69] continued the development of a molecular-level simulation of the FCC process based on the SU-BEM framework. They consciously selected a pathway-level kinetic modeling approach over a more detailed mechanistic-level one, justifying this simplification as necessary for the practical simulation of heavy oil feedstocks. Their resulting model, comprising 3652 molecules and 8202 reactions, was successfully integrated into a full process model of a riser-type FCC unit, demonstrating acceptable accuracy in predicting product yields, gasoline composition and key bulk properties, alongside molecular distributions. Guan et al. [332] showed how the established framework of SU-BEM was being refined with larger libraries and smarter algorithms to create faster, more accurate models with direct industrial applicability (blending of diesel). Xu et al. [384] used SU-BEM methodology to represent molecules and automate the generation of a detailed reaction network of ethane/propane steam cracking to simulate a cracking furnace in a refinery in China. A molecular-level reaction kinetic model coupled with a mathematical model of the cracking furnace process was developed. Accurate simulations of the composition, temperature, and pressure of the cracking gases were achieved by fitting reaction kinetic parameters to industrial data corresponding to five different outlet temperatures. The predicted yields of main products were in a good agreement with the industrial ones, validating the model adequacy. Sihang et al. [82] availed the SU-BEM framework, comprising 34 structural units divided into core and side structural units, to construct a molecular composition model of petroleum to resemble crude oil physical properties. According to the authors the crude oil experimental data allowed them to identify 98 representative molecular cores, involving 36 hydrocarbons and 62 heteroatomic cores, which by utilization of SU-BEM framework enabled building a crude oil molecular library consisting of 6524 characterized molecules. The developed model predicts very well the elemental composition of five crude oils with relatively close elemental composition. However, the physical properties boiling point and viscosity were not predicted very well.

The improvement in the SU-BEM framework is currently directed towards automated isomer generation to enrich structural units, graph-theoretic pruning of sparse reaction networks, and machine learning surrogates to reduce parameter loads [41].

#### 2.1.6. Other Approaches

Fernandes et al. [71] proposed a new framework based on the use of two matrices: (1) molecule as a graph (MAG) that contains detailed information about the bonds between atoms in a molecule; (2) an extended version of structure-oriented lumping (SOLex) that employs 44 structural increments organized into vectors. The joint employment of MAG and SOLex enable taking advantage of the flexibility of SOLex as well as the detail of MAG, allowing for the rapid collection of detailed information about molecules. This new framework was developed with the intention of being used for hydrocarbons from petroleum and biomass sources, as well as for various types of molecules, providing a flexible and efficient way to process molecular information. The proposed molecular modeling platform is compatible with other methods for property calculations such as group contribution and molecule drawing, converting to the SMILES language, etc.

Bi and Qiu [291] proposed a novel optimization-based method for the molecular reconstruction of naphtha founded on a more effective PDF that incorporates regional specificities and operational uncertainties using a self-adaptive cloud model. This PDF was then used within several algorithms (genetic algorithm (GA), PSO, SA) and demonstrated that the hybrid GA-PSO algorithm provided superior performance (average weight percentage deviation of 0.1625 and properties’ relative error below 5%) and computation time of under 34.2 s.

Yang et al. [297] benchmarked and compared different machine learning models for molecular property prediction. They proposed a novel graph convolutional neural network (GCN) that operates on a hybrid molecular representation, combining learned features from the molecular graph structure with expert-crafted molecular descriptors. They demonstrated that this model consistently matched or outperformed previous models based solely on fixed fingerprints or other graph architectures on a range of public and proprietary datasets. A key innovation is the use of convolutions centered on bonds instead of atoms, which is highlighted as a simplification that avoids unnecessary loops in the algorithm, potentially leading to more efficient computation.

Mei et al. [272] elaborated a novel molecular reconstruction method that consists of two Bayesian regression models for bulk properties’ prediction and molecular reconstruction. They proposed a two-step molecular reconstruction method. The first one includes building a likelihood function of the bulk properties from the molecular compositions by Bayesian parametric computation and a new mixing rule that uses a general characteristic function that features a higher accuracy than classic linear mixing rules. The second step consists of deriving the Bayesian estimation of the molecular compositions with a novel prior distribution of the molecular information obtained by supposing that all components in the blend are sampled identically and independently under Gamma distributions [272].

Guan and Zhang [110] developed a novel fast solving method, coined as presampling nearest neighbors-multiple search (PNN-MS), that drastically reduced (by a factor of 20–50) the computational time required for molecular reconstruction. They achieved this acceleration in the molecular reconstitution process by building a preconstructed virtual composition database and subsequent quadratic programming (SQP) to adjust the initial values. The authors bypassed the use of global optimization algorithms to solve the complex inverse problem (fitting model parameters to match bulk properties) and leveraged a preconstructed virtual oil database. When characterizing a new target oil, the method first identifies virtual oils with similar bulk properties from this database and uses their preoptimized model parameters as high-quality initial guesses. With an excellent initial guess provided by the database, the model can then utilize much faster local optimization algorithms to converge to the optimal solution for the target oil. Guan and Zhang [110] established that the dataset with the virtual oil number of 1,000,000 is appropriate to derive a high-accuracy molecular modeling. The solutions obtained feature higher accuracy, likely because the method starts closer to the global optimum, reducing the risk of convergence to local minima. It is important to note that this method is not a new reconstruction framework itself (like SOL or MTHS) but rather an efficient solving strategy that can be applied to existing compositional models, such as the SU-BEM method developed by the authors’ group. They applied the new fast solving method to reconstruct the molecular composition of diesel and vacuum gas oil. The computation time of the PNN-MS approach was reported to be less than 2 min (Intel Core i7 (>5 CUP), 16 GB memory, and 64-bit Windows system).

Guan et al. [116] introduced a new framework for reconstructing the molecular composition of vacuum gas oil on the base of HRMS as a reference and the dual-objective optimization strategy developed earlier by the same research group (that of Linzhou Zhang) [112]. The advance in the semi-quantitative molecular characterization based on HRMS of petroleum oils [39] allows detecting about 8000–10,000 molecules in VGO. On the base of the DBE ambit and the molecular formulas identified by HRMS the basic molecular structures were defined. Afterwards homologous series were obtained by addition of side chains to the molecular cores and a predefined molecular library was generated. In contrast to the typical predefined molecular libraries [151,210] founded on virtual molecules the method of Guan et al. [116] offered a molecular library which contains almost all detectable molecules. In addition, representative isomer structures were added to the library, such as n-paraffins and isoparaffins, and the VGO molecular library consisted of 12,138 molecules. The molecular reconstitution of molecular composition of VGO proposed by Guan et al. [116] first defines the molecular library and PDF combination, then determines the value range of optimization parameters and generates the initial guesses of optimization variables. On this basis, the genetic algorithm (GA) was used to optimize the molecular fractions. When the bulk properties and molecular fractions of the compositional model are consistent with the experimental data and HRMS data, the final solution was obtained. Three compositionally different VGOs were tested for the application of the new molecular modeling framework, showing a good agreement with experimental bulk properties and the molecular distributions of different compound types determined by the HRMS data. The calculated molecular composition of the studied VGO samples showed good repeatability, suggesting its further application for heavy oil processing and geochemical research.

### 2.2. Advances in Petroleum Characterization Methods Supporting the Progress in Molecular Modeling of Petroleum

A remarkable advance in the quantitative molecular characterization of petroleum oils is presented in the study by Li et al. [38], who studied the gap between qualitative petroleomics and quantitative analysis by combining multiple ionization techniques (positive/negative-ion electrospray ionization, positive-ion atmospheric pressure photoionization) and chemical derivatizations. While high-resolution mass spectrometry (specifically Orbitrap MS) has been used for qualitative analysis (petroleomics), a major challenge has been its lack of quantitative data. Li et al. [38] addressed this directly by presenting a strategy for obtaining quantitative analysis of a heavy petroleum fraction (FCC decant oil). Their approach allowed them to assign and quantify over 7000 molecules across 20 different class species (e.g., aromatic hydrocarbons, nitrogen-containing compounds). The results are presented in weight percentages (wt.%) and parts per million by weight (wppm), providing a truly quantitative dimension previously difficult to achieve. For example, they quantified aromatic hydrocarbons at 49.6 wt.% and specific molecules across a concentration range from 2.0 × 10^−3^ to 2.1 × 10^4^ wppm. The quantitative molecular composition data provided by this method is precisely the kind of high-quality, detailed input required to construct more accurate molecular models and perform mechanistic process simulations.

Li et al. [30] introduced a methodological framework for using a single technique—electrospray ionization high-resolution mass spectrometry (ESI-HRMS)—to characterize a much wider range of petroleum compounds than traditionally possible. Conventionally, ESI is limited to polar heteroatomic compounds (e.g., nitrogen-containing species). The novelty in the study of Li et al. [30] lies in their approach to enable ESI to analyze traditionally “invisible” components: hydrocarbons (saturates and aromatics) and sulfur-containing compounds (sulfides and thiophenes). The key advancement is the shift from qualitative fingerprinting to semi-quantitative analysis. The authors achieved this by integrating the molecular-level data from ESI-HRMS of four different vacuum gas oils with bulk property data (e.g., overall sulfur content from elemental analysis). This allows them to calculate the mass fraction and approximate molecule number for over 5000 molecules across 16 compound classes. They validate their approach by comparing the H/C ratio calculated from their molecular composition with the H/C ratio from direct elemental analysis, finding acceptable agreement. The semi-quantitative molecular composition derived by this novel analytical technique allowed them to define a set of representative molecules for each VGO with specific structures.

Zhang et al. [40] moved beyond traditional bulk property analysis such as SARA analysis to provide a nuanced molecular picture. They explicitly mentioned and utilized high-resolution analytical methods that have become more accessible and standardized in recent years. These include: comprehensive two-dimensional gas chromatography (GC × GC) used to resolve thousands of compounds that would appear as an “unresolved hump” in traditional 1D-GC, providing a more detailed view of the hydrocarbon skeleton; FT-ICR MS coupled with soft ionization techniques like electrospray ionization (ESI), which allows for the ultrahigh-resolution characterization of polar compounds (e.g., nitrogen, oxygen, and sulfur-containing species) that are not amenable to gas chromatography (GC) analysis; chemical derivatization methods like methylation/demethylation to selectively separate and analyze specific compound classes (e.g., sulfides vs. thiophenes), making non-polar compounds amenable to ESI-MS analysis. This represents a sophisticated approach to species quantification, explaining the reason why Karamay heavy oil exhibits a high viscosity index, which lies in the high concentration of compounds with long side chains and low naphthenic skeletons. The techniques described are crucial for providing the detailed, quantitative input data required for modern molecular reconstruction and modeling efforts.

Ye et al. [318] developed a molecular level model for delayed coking using a sophisticated feedstock characterization framework. It consisted of supercritical fluid extraction and fractionation (SFEF) to separate the residue feedstock into narrow fractions and SARA fractionation of the narrow SFEF fractions using ASTM D4124 [444] into subfractions of saturates, aromatics, resins, and asphaltenes. Mass spectrometry (MS) was availed to distinguish the saturates as paraffins and naphthenes with a number of naphthenic rings between 1 and 6. MS was also employed to differentiate the aromatic fractions into aromatics with a number of aromatic rings between 1 and 5. The resins and asphaltenes were analyzed using NMR to obtain the average structure parameters according to the Brown–Ladner method [445]. In this way the delayed coking model developed by Ye et al. [318] in 2021 in contrast to that of Tian et al. [225] established in 2012 discerned with the much more sophisticated feedstock characterization framework. Tian et al. [225] used only residue feedstock properties, which characterized the whole residue, such as density, Conradson carbon content, SARA composition, elemental composition, and average molecular weight, which could not provide a detailed feedstock characterization as did the framework used by Ye et al. [318]. In both studies by Tian et al. [225] and by Ye et al. [318], the SOL method was used to build the molecular model of the residue feedstock chemical composition. In contrast to the study by Tian et al. [225], where 22 structure vectors were availed, in the investigation by Ye et al. [318] 24 structure vectors were employed to reconstruct the residue feedstock. The molecular model by Ye et al. [318] consisted of 2944 molecules versus 7004 in the model by Tian et al. [225]. Nevertheless, the more sophisticated analytical framework of the feedstock applied in the study by Ye et al. [318] along with the use of two more structure vectors, although using the same algorithm as that of Tian et al. [225], provided calculation errors of the feed properties an order of magnitude lower than that of Tian et al. [225]. This highlights the meaning of the advanced oil characterization for improvement in the molecular modeling of petroleum, which obviously outweighs the significance of the number of molecules used to reconstruct the petroleum residue. Concerning the prediction of delayed coking yields both studies reported almost the same accuracy of prediction, suggesting that the characterization of feed does not have such a strong effect on the forecasting of product yields. However, in the study by Ye et al. [318], the composition of coker naphtha and diesel fuel was reported, while in the study by Tian et al. [225], no information on the composition of these products was provided. It is worth noting here the very accurate prediction of the distillation curve obtained in the study by Ye et al. [318]—a fact that has not yet been observed in the studies published up to 2021, confirming that the analytical framework proposed by Ye et al. [318] is the most informative by this time.

## 3. Discussion

Molecular modeling technology can be viewed as a tool to improve our understanding of petroleum itself and refining technology, as it incorporates as many fundamental principles as possible to quantitatively describe phenomena in their greatest detail. Like most models, the molecular composition models of petroleum can also be seen as an imperfect approximation of the real system [446]. This can be explained by the extremely high complexity of the diverse petroleum fluids, which cannot always be sufficiently well described by the algorithm used and the analytical procedure applied. For example, in the study of Campbell et al. [202], who employed the same SR framework to reconstruct four vacuum residues (Hondo, Maya, Arabian Light, and Arabian Heavy), an excellent fit for the kinetic parameters of thermal cracking of Arabian Light and Maya residues and a poorer reaction model fit for the Hondo and Arabian Heavy residues were obtained. A possible explanation for the poorer modeling results for the Hondo and Arabian Heavy residues may be considered the poorer structural fits for these two residues, i.e., the structural model deviations propagated uncertainty in the reaction model. This highlights the great importance of the chemical nature of petroleum fluids modeled at the molecular level. The review articles dealing with the molecular modeling of petroleum appearing so far (Ren et al. [43], Wang et al. [47], and Chen et al. [49]) were focused mainly on the progress achieved in the molecular modeling methods. Less attention was paid to the subject of the modeling—the chemical composition of petroleum. In reviewing the literature on the molecular reconstruction of crude oil, we found that the compositions of the crude oils studied, which were modeled, did not cover the full range of variation in the chemical compositions of crude oils worldwide. The studies of Kheirollahi [447], Hinkle et al. [448], and Shishkova et al. [449] present a good example of the limits of variation of crude oil chemical composition expressed by the contents of SARA fractions and density. The data in Table 3 can be considered representative of the limits in the variation of density and SARA fraction contents of the crude oils around the globe. A correlation analysis was performed to examine the relationship between density and the content of the different SARA fractions, as density has been a target in the optimization algorithms of the molecular reconstruction giving the best result [416]. The results of a correlation analysis of the data in Table 3 are given in Table 4.

(1) $$Hydrogen\;content\;of\;crude\;oil\;(wt.\%)=-14.802\times D_{20}+25.575\;R\;=\;0.99$$

**Figure 10:**
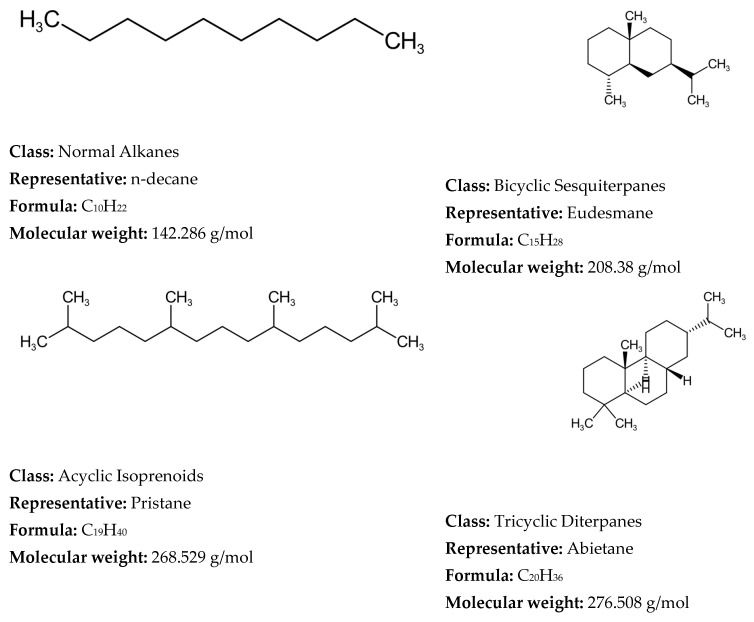

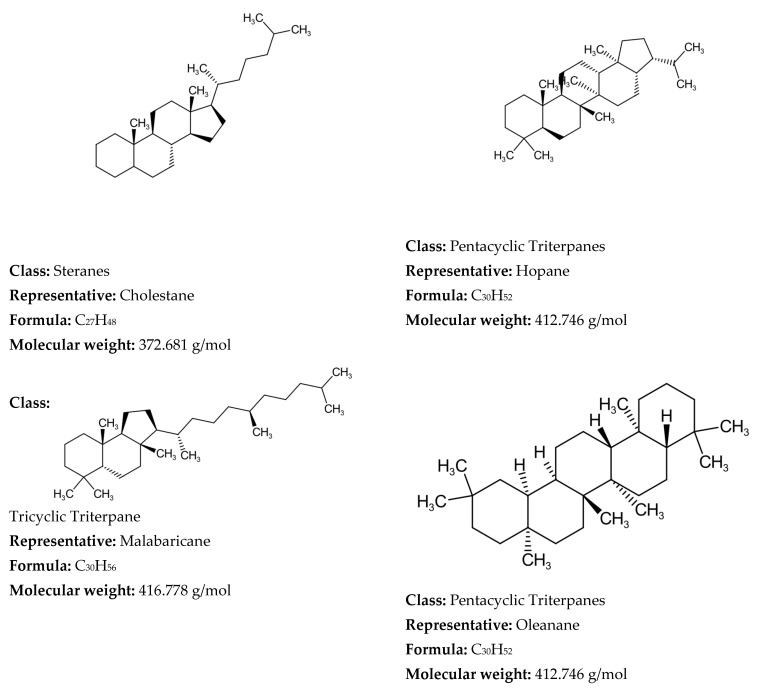
Representative structures of saturated hydrocarbons, found in crude oils. (Adopted from Overton et al. [450], Overton et al. [451], Adams et al. [452], Stratiev et al. [290]).

**Figure 11:**
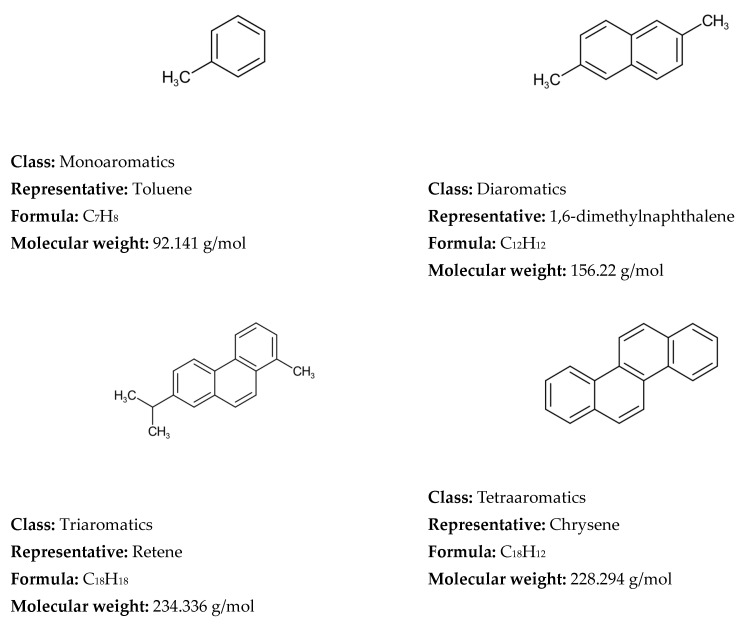

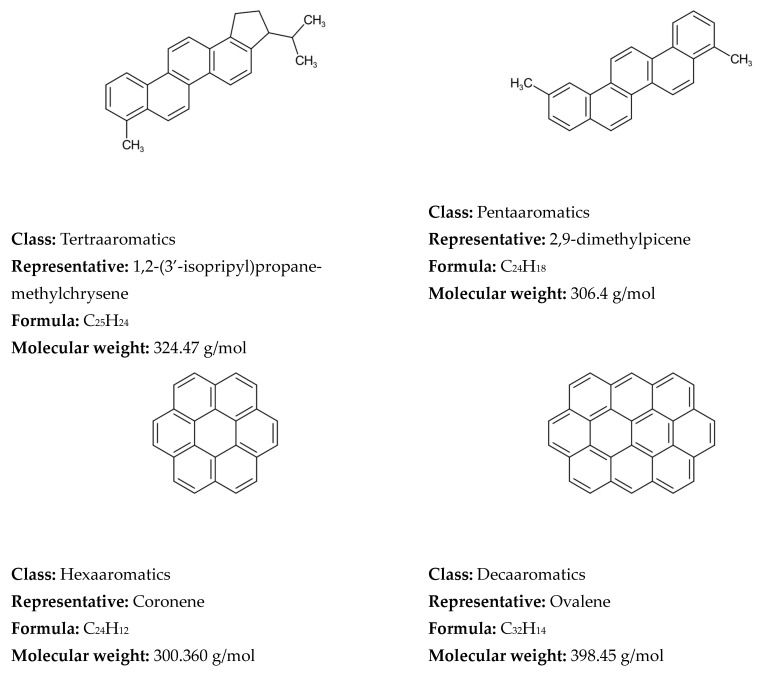
Representative structures of aromatic hydrocarbons, found in crude oils. (Adopted from Overton et al. [450], Overton et al. [451], Adams et al. [452], Stratiev et al. [290]).

**Figure 12:**
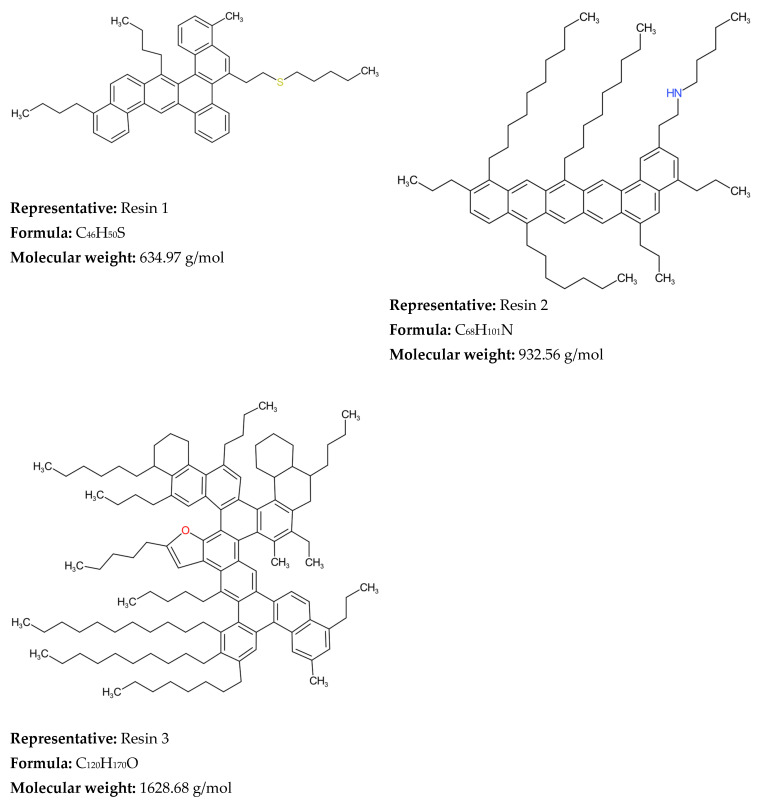
Representative structures of crude oil resin (polar) fraction. (Adopted from Overton et al. [450], Overton et al. [451], Yakubov et al. [453], El Nagy et al. [454], Stratiev et al. [290]).

**Figure 13:**
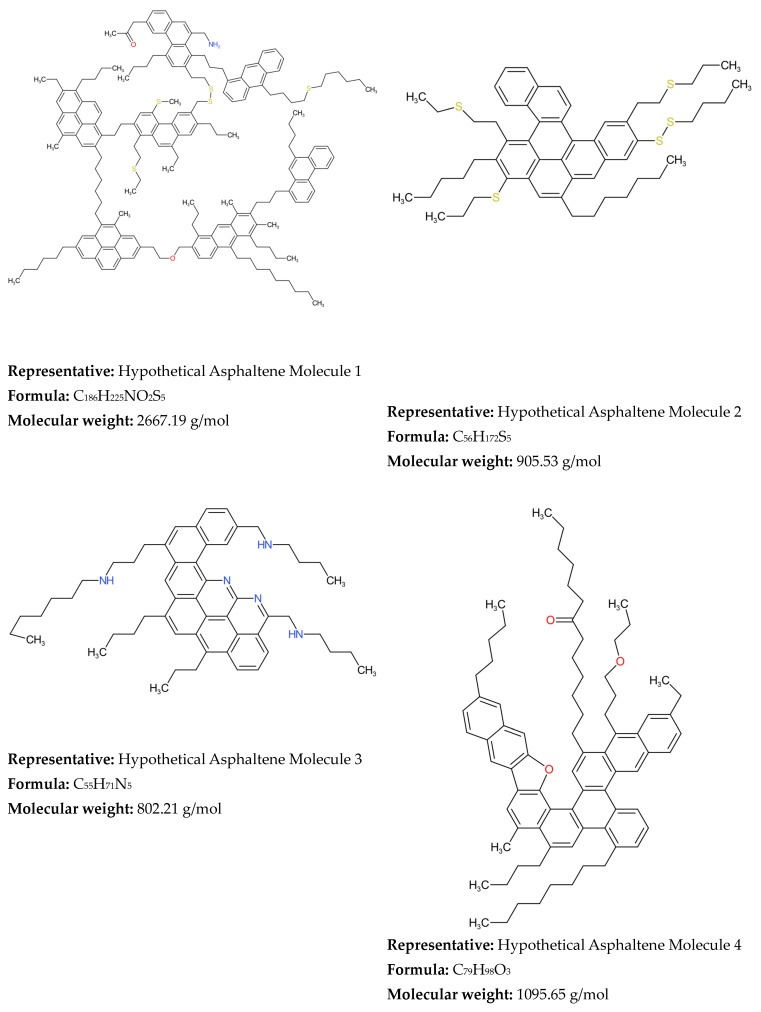
Representative structures of crude oil asphaltene fraction. (Adopted from Overton et al. [450], Overton et al. [451], Zuo et al. [455], Stratiev et al. [290]).

**Figure 14:**
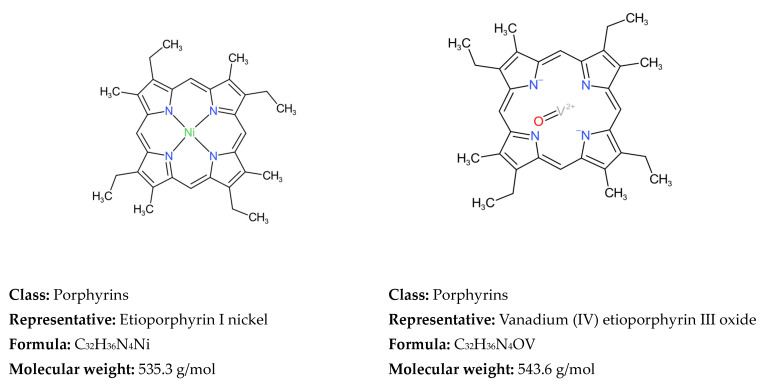
Representative structures of crude oil porphyrins. (Adopted from Overton et al. [447], Overton et al. [450]).

The data in Table 4 do indeed confirm that density is strongly correlated with the content of saturated hydrocarbons and asphaltenes, as well as a slightly weaker, but still strong, correlation with the resin content. The possible structures, which are present in the different SARA fractions of petroleum, are summarized in Figure 10, Figure 11, Figure 12, Figure 13 and Figure 14.

**Figure 15:**
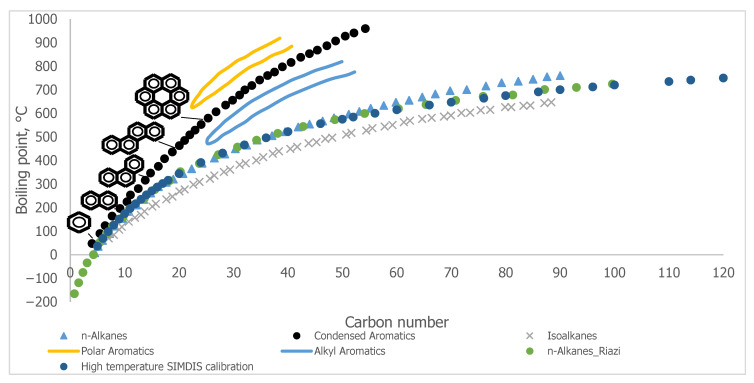
Relationship between the number of carbon atoms and the boiling point of the various hydrocarbons and their polar derivatives found in crude oil (modified from Ramirez-Corredores [456] and Riazi [442]).

The distribution of different molecular structures based on the number of carbon atoms in the molecule influences the distillation curve of crude oil, as shown in Figure 15.

**Figure 16:**
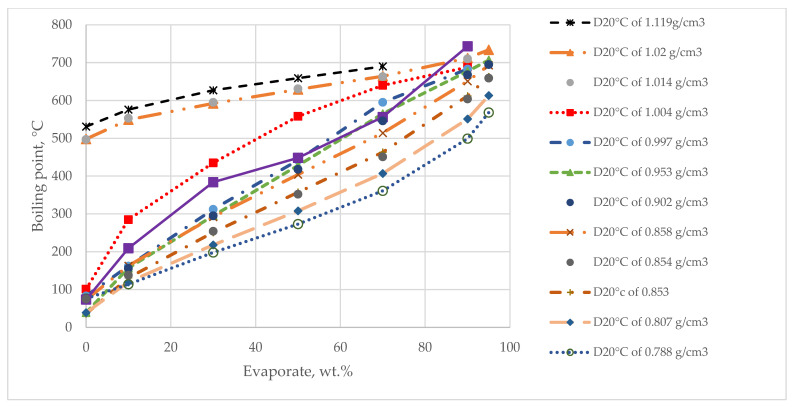
HTSD of crude oils whose density and SARA composition are shown in Figure 6.

Figure 16 presents data of high-temperature simulated distillation (HTSD, ASTM D7169) of the crude oils from Figure 6.

It is evident from the data in Figure 16 that distributions of components according to the boiling points of the 12 crude oils are as different as their SARA compositions.

The data in Table 3 and Figure 16 show that the diversity in the group hydrocarbon composition (SARA), as well as the boiling point distribution and therefore the molecular weight distribution, is so great that the proof of the ability of a given molecular modeling technique to adequately mimic the actual molecular composition of crude oil should be accepted when it is validated across the entire range of variation of density, SARA composition and boiling point distribution. Zhou et al. [336] discussed the catalytic cracking of Daqing crude oil, whose properties are presented in the data of Figure 6 and Figure 16. Zhang et al. [42] mentioned that they examined their novel method SOHSL-CM by availing 42 sets of crude oil data without providing any information about these crude oil data. Sihang et al. [82] predicted the physical properties of five crude oils whose hydrogen content varied between 11.98 and 13.65 wt.%. Xie et al. [417] modeled the molecular chemical composition of 22 crude oils whose density varied between 0.830 and 0.970 g/cm^3^ and distillation characteristics oscillated for their initial boiling point between 273 and 398 °C and 50% evaporate temperature in the range 537–765 °C. All these investigations indicate that the full range of possible variations in the properties of crude oils being modeled, as illustrated in the data of Figure 6 and Figure 16, was not covered. This finding suggests that the future investigations of crude oil molecular reconstitution should be directed to cover all possible variations in petroleum bulk properties and especially those petroleum oils which have a higher concentration of resins and asphaltenes. These petroleum fractions, as evident from the data in Figure 12 and Figure 13, are the most complex and therefore the most difficult to model.

**Figure 17:**
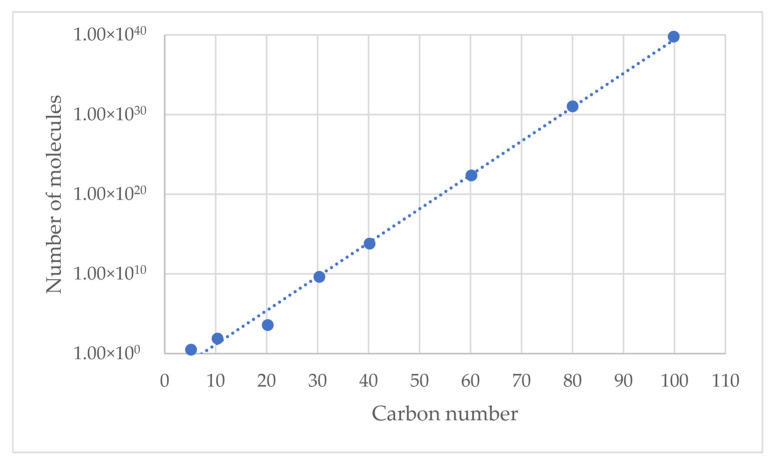
Number of molecules in crude oil as a function of their carbon number. (Adapted from Broeke [457]).

Broeke [457] calculated the number of potentialorganic substances for such a broad carbon number range to be in 10^60^. Figure 17 shows the dependence of number of molecules in crude oil on the carbon number of the constituents.

Petroleum scientists consider that the number of different molecules in petroleum is formidable but not as astronomical as predicted by simple combinatorics [300]. The number of molecules in petroleum oils used to reconstruct their molecular chemical composition was reported in several studies to affect the accuracy of property predictions [48,130], but this number ranges in the order of 10^4^–10^5^ [130,137]. The greatest number of molecules used to reconstruct petroleum oil (Venezuelan vacuum residue) in all reviewed studies is 400,000 [113]. Despite the use of 400,000 molecules, built on the base of about 600 structure attributes, by Zhang et al. [113] the calculated SARA composition and simulated distillation curve featured a lower accuracy compared to the calculations of Yang et al. [81] who availed 6900 molecules and another molecular modeling framework (SOL). This comparison implies that the number of molecules is not as critical as the modeling strategy used to adequately reconstruct the molecular composition of petroleum. The research of Yang et al. [81] proposed a molecular modeling strategy that seems easy to apply in terms of the availability of SARA and element composition data along with simulated distillation and to report accurate calculations with a tractable number of molecules. However, it needs to be validated for vacuum residual oils featuring high resin and asphaltene content such as Boscan VR (resins + asphaltenes of 57.6 wt.%), Vasconia VR (47.9 wt.%), and Castilla VR (54.0 wt.%), whose hydroprocessing is very challenging [438] (see the data in Table 4). Table 4 summarizes the SARA and element composition, with high-temperature simulated distillation of vacuum residues, from the database of the research laboratory of LUKOIL Neftohim Burgas, which could be considered representative of the boundaries of property variation (minimum and maximum), except the molecular weight, for vacuum residues from all over the world. The molecular weight of petroleum vacuum residues can vary between 600 and 1440 g/mol as reported in [81,458] and this should be taken into account in the process of molecular reconstruction. Vacuum residues reconstructed in the studies of de Oliveira et al. [61], Campbell and Klein [137], Zhang et al. [113], Yang et al. [81] and others reviewed in this work do not cover the whole range of variation of vacuum residue characteristics as shown in Table 5. Therefore, additional molecular reconstruction studies are needed to embrace the whole range of property variation of vacuum residues and prove the applicability of the molecular modeling frameworks. The same is valid for the vacuum gas oils reconstructed in the research of Wang et al. [335], Ren et al. [43] and other research reviewed in this article, which do not cover the full compositional diversity for VGO as reported in the research of [459].

**Figure 18:**
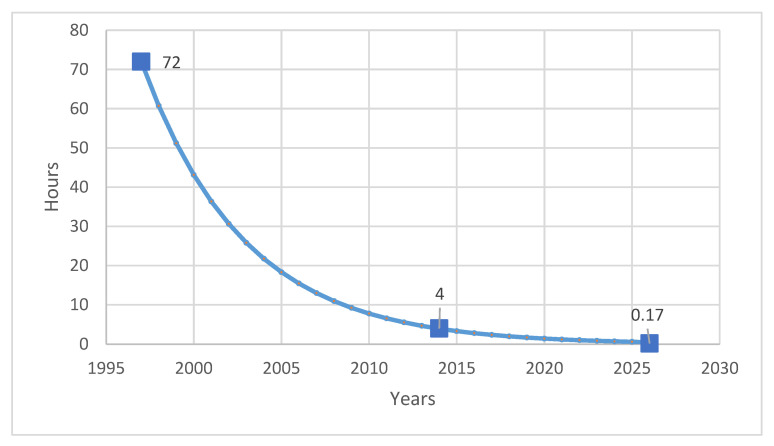
CPU time evolution for molecular reconstruction of vacuum residue over the years.

Most molecular modeling frameworks used in the various studies on the recovery of oil and its fractions during the considered period of 1989–2025 and beginning of 2026 show that they are capable of reconstructing all types of petroleum fluids (Table 6), despite the fact that their original versions have shown some limitations, mainly for heavy oils. As was displayed in Figure 4 the SOL method was the most frequently explored, followed by SR, hybrid, and MTHS (Figure 4). MTHS was predominantly used for modeling of light oils, which restricted its usage [48]. However, the research of Song et al. [41] opened a new perspective for the MTHS approach, showing that it can be used for reconstruction of not only light oils but also heavy petroleum fractions, reporting extremely short computation time (between 3 and 36 s). Over the years, the applied molecular reconstruction frameworks demonstrated significant progress in terms of range of application, speed of computation, and accuracy of prediction. It is difficult to rank any of the investigated methodologies considering the achieved progress, although there is observed predominance of the SOL method utilized in the molecular reconstitution of petroleum. Each methodology investigated has its advantages and disadvantages, depending on the required application, but what is desired is a representation that preserves the maximum amount of molecular information while maintaining the necessary simplicity in computational manipulation. Regarding the progress in the speed of molecular reconstruction, Figure 18 illustrates the huge improvement in the applied algorithms, hardware and modeling framework from 1997 to early 2026, allowing a 423-fold reduction in CPU time for the reconstruction of the most difficult to model petroleum fraction—vacuum residue. Different optimization algorithms (GA [81,116], SA [225,413], SQP [110,417], ANN + GA [265], non-linear least squares optimization [41,81], PSO [291], GA-PSO [291], Bayesian likelihood-free inference [14]) have been applied over the years.

Their successful performance was found to depend on the specificity of the modeling strategy and it cannot be deduced that a given algorithm exhibits the best performance in all petroleum molecular reconstitution studies. The improvement in the molecular reconstitution computation time observed lately enables using the reconstructed molecular composition of petroleum in process modeling and optimization [48,95,96].

Despite significant advances in molecular reconstitution methodologies, their integration with commercial process simulation software remains challenging. Most widely used simulators such as Aspen HYSYS, Aspen Plus and others continue to rely primarily on pseudo-component approaches for petroleum characterization [77,86]. This discrepancy creates a gap between the detailed molecular information generated by reconstitution models and the simplified representations employed in process simulation. Research in this field showed that the gap could be bridged by three approaches: (1) molecular reconstitution is performed outside the process simulator environment and the resulting molecular compositions or pseudo-components with enhanced structural information are then imported into the simulator as user-defined components [246,284]; (2) a hybrid approach combines traditional pseudo-component generation with molecular information. This strategy balances computational efficiency with molecular detail where it matters most for reaction kinetics [101]; (3) development of custom reactor models that directly utilize molecular reconstitution outputs, bypassing the limitations of standard simulator components. These models often employ reduced reaction networks derived from detailed molecular kinetics [69,341]. The integration of molecular reconstitution models with process simulators faces several technical challenges such as computational overload, data consistency, reaction network complexity and lack of standardization. Recent developments in software architecture, particularly the adoption of CAPE-OPEN standards and the emergence of open-source simulation platforms like DWSIM, offer promising avenues for more seamless integration of molecular reconstitution approaches into the process simulators [88,89].

## 4. Conclusions

Petroleum is a highly complex mixture of hydrocarbons and heteroatom-containing organic substances whose molecules may theoretically reach 10^60^. Despite the doubts expressed by some petroleum scientists that the number of different molecules in crude oil is not as astronomical as calculated by combinatorics, it is recognized that their number is formidable. Thus, the molecular reconstruction of petroleum is a highly challenging task. Since 1989, when this methodology was first introduced, molecular modeling of the chemical composition of petroleum has made significant progress. Initially (the period 1989–2024), the molecular modeling of petroleum consisted of constructing of virtual molecules on the base of petroleum characterization data such as bulk properties, simulated distillation, NMR, elemental and SARA composition, and molecular weight without reference to molecular-level characterization information. However, with the advance of analytical methods and the appearance of semi-quantitative petroleum characterization in 2024 it became possible to incorporate molecular-level characterization information into the modeling process of the compositional model. In this way, the molecular compositional models became as close as possible to the actual composition of petroleum. Diverse molecular modeling frameworks were introduced such as SR, SOL, MTHS, and SU-BEM and hybrids like SR-REM, SOL-Monte Carlo, SOL-ANN, SOHSL-CM. The optimization techniques used within these frameworks such as non-linear least squares optimization, simulated annealing, genetic algorithm, sequential quadratic programming, and others along with the advanced hardware enabled the molecular reconstruction process to be done in a few days in the 1990s down to a few seconds in 2025. All this made it possible to incorporate molecular reconstruction models into refining process models and optimize refining performance. The progress achieved in the molecular reconstitution of petroleum enables its application in the elaborate process of crude oil selection in petroleum refinery and for geochemistry studies.

All molecular reconstitution studies reviewed for the period 1989–2025 and early 2026 show that they do not embrace all the ranges in oil properties and compositions observed in crude oils worldwide. Future research is therefore expected to be directed at validating the established molecular modeling frameworks with petroleum oils whose range of property variations covers all observed limits to prove their general applicability. The challenge of integrating molecular oil reconstitution models with process simulation software should also be addressed in future research.

## Figures and Tables

**Table 1:** Summary of publications dealing with molecular modeling of crude oil and oil fractions.

Year of Publication	References	Year of Publication	References
1989	[98,99,100]	1990	[117]
1991	[118,119]	1992	[120,121,122,123]
1993	[124,125]	1994	[102,126,127,128,129,130,131]
1995	[132,133]	1996	[134,135,136]
1997	[137,138,139,140,141,142]	1998	[143,144,145,146,147,148,149]
1999	[150,151,152,153,154,155]	2000	[156,157,158]
2001	[159,160,161,162,163,164,165,166]	2002	[167,168,169,170,171]
2003	[172]	2004	[173,174,175,176,177,178]
2005	[51,57,179,180,181,182,183,184,185,186,187]	2006	[188,189,190]
2007	[191,192]	2008	[64,193,194,195,196,197,198,199]
2009	[200,201,202,203,204,205,206]	2010	[207,208,209,210,211,212,213,214,215,216]
2011	[62,217,218,219,220,221,222,223]	2012	[224,225,226,227,228,229,230]
2013	[13,104,108,231,232,233,234,235]	2014	[107,113,236,237,238,239,240]
2015	[50,241,242,243,244,245,246,247,248]	2016	[249,250,251,252,253,254,255,256,257,258,259,260,261,262]
2017	[60,74,263,264,265,266,267,268,269,270,271,272,273,274,275,276]	2018	[70,93,277,278,279,280,281,282,283,284,285,286,287]
2019	[37,43,55,66,67,114,288,289,290,291,292,293,294,295,296,297,298,299,300,301,302,303,304,305,306,307]	2020	[46,308,309,310,311,312,313,314,315,316,317]
2021	[68,94,115,318,319,320,321,322,323,324,325,326,327,328,329,330,331]	2022	[42,59,69,72,110,332,333,334,335,336,337,338,339,340,341,342,343,344,345,346,347,348,349]
2023	[45,47,54,112,350,351,352,353,354,355,356,357,358,359,360,361,362,363,364,365,366,367,368,369,370,371,372,373]	2024	[9,14,49,63,65,82,87,101,374,375,376,377,378,379,380,381,382,383,384,385,386,387,388,389,390,391,392]
2025	[41,48,71,78,81,109,111,393,394,395,396,397,398,399,400,401,402,403,404,405,406,407,408,409,410,411,412,413,414,415]	2026	[10,116,416,417,418]

**Table 2:** Comparison of molecular reconstitution approaches for molecular modeling of petroleum.

Approach	Key Characteristics	Typical Applications	Advantages	Limitations
SR	Monte Carlo sampling from probability distributions of structural attributes	Heavy fractions, residues, asphaltenes	Handles complex mixtures with uncertain composition; suitable for heavy ends	Restricted molecular library (absent compounds cannot be reconstituted) [43]; high computational cost [321]; high variability as a result of random sampling prevents convergence [14]
SOL	Molecules represented as vectors of structural increments	FCC, hydrocracking, hydrotreatment, catalytic reforming, coking	Balanced detail and computational efficiency; widely adopted in industry	Reactive intermediates ignored, making mechanistic kinetics intricate; high dependence on data from sophisticated analytical techniques; combinatorial explosion of vector libraries; artificial sampling constraints in stochastic variants; simplified representation of molecular structures and isomer unclarity, as vectors cannot discern stereochemistry [41]
MTHS	Two-dimensional matrix (carbon number × homologous series)	Light and middle distillates, naphtha, reforming, hydrotreating, and blending	Systematic organization; facilitates property prediction	Limited for very heavy fractions for building molecular libraries; highly dependent on high-resolution analytical data [217]
REM	Maximizes information entropy under analytical constraints	Various fractions from naphtha to VGO and vacuum residue	Mathematically rigorous; integrates diverse data sources	Optimization can be computationally demanding [62]; strong dependence on the initial set of molecules; very sensitive to the predefined initial set of molecules and the properties of the mixture calculated on their basis, when they are too far from the analytically measured values [174]
SU-BEM	Combines structural units with bond-electron matrices	Heteroatom-containing compounds, hydroprocessing	Captures electronic effects on reactivity	Complex implementation; requires specialized expertise; structural units are not able to discern all isomers, leading to compositional unclarity; BEM construction and handling demand expert coding proficiency; huge reaction network requiring high computational costs [67,68]

**Table 3:** SARA analysis and density data of different crude oil samples from around the world.

Density at 20 °C (D_20_), g/cm^3^	Saturates, wt.%	Aromatics, wt.%	Resins, wt.%	Asphaltenes, wt.%	Hydrogen Content, wt.% **
1.119	4.0	17.0	37.0	43.0	9.01
1.021	28.3	39.1	11.4	21.2	10.46
1.014	22.5	40.2	16.4	20.3	10.56
1.004	27.3	38.5	15.3	19.2	10.71
0.997	24.8	41.0	17.9	16.3	10.82
0.953	34.2	36.3	12.9	16.6	11.47
0.902	36.7	39.1	12.0	12.2	12.22
0.858	76.7	15.0	3.6	2.7	12.88
0.854	67.3	26.5	3.3	2.9	12.94
0.853	73.4	15.7	4.4	6.2	12.95
0.807	94.8	3.4	0.8	1.0	13.62
0.788	74.8	24.5	0.7	0.1	13.91
0.863 *	70.1	19.8	9.5	0.0	13.55

Note: * The data in the last row are related to Daqing crude oil [418]. ** Hydrogen content of crude oil was calculated using Equation (1) (in-house method).

**Table 4:** Correlation matrix of the data in Figure 6.

	Density	Saturates	Aromatics	Resins	Asphaltenes
Density	1				
Saturates	−0.95	1			
Aromatics	0.51	−0.70	1		
Resins	0.91	−0.88	0.30	1	
Asphaltenes	0.96	−0.91	0.36	0.94	1

**Table 5:** Vacuum residues obtained from different crude oils, comprising the possible boundaries of property variation.

VR from Crude Oil (360–540 °C)	Arab Med	Arab Heavy	Arab Light	Basra Light	Basra Heavy	Kirkuk	El-Bouri	Rhe-Moura	CPC	Azeri Light	Vara-Ndey	Boscan	Alba-Nian	Prinos	Tempa Rossa	Sib Light	Urals	Vasconia	Castilla
Density at 15 °C, g/cm^3^	1.031	1.04	1.029	1.052	1.071	1.054	1.05	1.041	0.94	0.967	0.99	1.078	1.094	1.108	1.12	0.993	0.997	1.057	1.067
Concarbon content, wt.%	20.7	23.6	18.7	23.8	28.9	25.2	25.5	23.7	12	9.5	15.1	27.8	31.4	32.8	34.3	14	17.5	31.1	34.0
Sulfur, wt.%	5.4	5.8	4.9	5.9	7.1	5.9	3.3	1.8	2.1	0.5	1.7	6	8.7	9.14	9.3	1.58	3	2.29	3.62
Nitogen, wt.%	0.29	0.437	0.368	0.418	0.408	0.518	0.489	0.391	0.311	0.568	0.28	0.767		0.5	0.531	0.42	0.506	0.646	0.955
Saturates, wt.%	9.25	6.16	11.32	7.92	7.40	9.52	14.69	15.69	39.63	34.94	31.06	1.55	1.20	4.61	0.75	21.15	19.77	9.80	12.20
Aromatics, wt.%	70.91	67.03	73.88	67.40	69.32	69.11	63.51	62.13	51.01	53.25	50.82	40.83	61.01	66.88	64.71	61.03	62.88	42.30	33.80
Resins, wt.%	11.01	13.59	8.98	14.41	13.42	10.20	10.15	11.40	7.02	10.67	13.08	23.39	12.88	11.14	10.87	13.38	11.67	24.20	23.20
Asphaltenes, wt.%	8.82	13.21	5.81	10.27	9.86	11.15	11.64	10.78	2.33	1.12	5.01	34.20	24.83	17.32	23.55	4.38	5.68	23.70	30.80
Hydrogen, wt.%	10.93	10.36	10.59	10.80	9.69	10.05	10.60	10.80	12.51	12.30	10.90	10.00	9.40	9.00	8.90	11.37	11.0	10.1	9.77
High-temperature simulated distillation (ASTM D 7169 [460]), °C																			
Initial boiling point	513	524	498	507	488	513	478	487	487	483	520	494	503	491	531	508	497	500	
10%	560	567	549	560	537	558	523	533	518	526	559	564	567	539	576	553	553	553	
30%	600	614	592	603	588	603	568	577	551	567	598	615	617	574	627	585	595	598	
50%	633	653	629	637	626	645	610	617	584	605	635	644	647	613	659	592	631	636	
70%	663	686	664	666		680			625	644	674	671	674	649	690	668	663	669	
90%			712	710							738	713	715			719	710	713	
95%			734									757	762					735	
Final boiling point	691	761	740	713	643	709	642	650	646	650	764	938	952	663	696	730	718	780	
Recovery,%	84.0	85.1	96.6	91.0	62.6	84.7	66.2	67.0	79.1	73.1	92.2	73.95	64.21	78.8	74.4	91.8	93.3	76.63	
MW, g/mol	722	791	709	721	672	749	636	663	606	675	759	729	729	611	758	620	739	714	

**Table 6:** Application of the diverse molecular modeling approaches for reconstruction of petroleum fluids.

	Reconstruction Method	SR	SR-REM	SOL	MTHS	PNN-MS	REM	SU-BEM
Petroleum fluid being reconstructed	Crude oil			[42,44,336,418]	[9]	[417]		
	Vacuum residues	Hondo, Maya, Arabian Light, Arabian Heavy, Venezuelan [113,202,247]	Buzurgan (Iraq), Russia (Ural), Mexico (Maya), Canada (Athabasca), Indonesia (Ardjuna and Duri), Congo (Djeno), Saudi Arabia (Arabian Medium) [61], Athabasca [104], Arabian Light [210]	Two Sinopec vacuum residues [230], Saudi: Diaz: Soros: Oman (2.6:2:0.2:5.2); Iran: Soros: Saudi: Diaz (0.5:1:6.5:2); Basra: Kuwait: Mundo (3.6:2.4:4); and Soros: Kuwait: Djeno (1.5:5.3:3.2) [225], Venezuelan [81]		[417]		
	Atmospheric residue			[267,329]	[41]			
	Vacuum gas oils	[252,321]	[335]	[150,228,329,370,385]	[41]	[110]		[67,68,69]
	Diesel	[187,193,208]	[173,174]	[408]	[203]	[110]		[67]
	Gasoline	[187,193]		[48]	[164]		[62,209]	[384]

## Data Availability

Data are contained within the article.

## Abbreviations

ACS	American Chemical Society
AFM	atomic force microscopy
AI	artificial intelligence
ANN	artificial neural network
API	American Petroleum Institute
APPI FT-ICR	atmospheric pressure photoionization Fourier transform ion cyclotron resonance
ASTM	American Society for Testing and Materials
BEM	bond-electron matrix
BO	Bayesian optimization
C	carbon
13C NMR	carbon-13 nuclear magnetic resonance
CasADi	computer algebra systems algorithmic differentiation
CatBoost	categorical boosting
CBR	case-based reasoning
CCR	continuous catalytic reforming
CCS	column chromatography separation
CME	composition model editor
CN	cetane number
CPU	central processing unit
D_20_	density at 20 °C
1D-GC	one-dimensional gas chromatography
DBE	double bond equivalent
DH	diesel hydrotreater
DMCD	data-mechanism complementary dominance
DNN	deep neural network
ESI	electrospray ionization
ESI-MS	electrospray ionization mass spectrometry
ESI-HRMS	electrospray ionization high-resolution mass spectrometry
FCC	fluid catalytic cracking
FCCGH	fluid catalytic cracking gasoline hydrotreater
FNN	feedforward neural network
FT-ICR MS	Fourier transform ion cyclotron resonance mass spectrometry
FT-IR	Fourier transform infrared spectroscopy
GA	genetic algorithm
GA-PSO	genetic algorithm–particle swarm optimization
GC	gas chromatography
GC × GC	two-dimensional gas chromatography
GC-FID	gas chromatography–flame ionization detector
GCN	graph convolutional neural network
GC × GC-TOF MS	two-dimensional gas chromatography with time-of-flight mass spectrometry
H	hydrogen
1H NMR	proton nuclear magnetic resonance
H/C ratio	hydrogen-to-carbon atomic ratio
HCO	heavy cycle oil
HPLC	high-performance liquid chromatography
HRMS	high-resolution mass spectrometry
IFP	Institut Français du Pétrole (French Institute of Petroleum)
InGen	Interactive Network Generator
IPOPT	interior point optimizer
kMC	kinetic Monte Carlo method
KME	kinetic model editor
KMT	kinetic modeler’s toolbox
LCO	light cycle oil
LFER	linear free energy relation
LPG	liquefied petroleum gas
m	independent variable used in modified gamma distribution
MAG	molecule as a graph
MALDI-TOF MS	matrix-assisted laser desorption ionization time-of-flight mass spectrometry
MC	Monte Carlo
ML	machine learning
MLR	multiple linear regression
mMTHS	modified molecular type homologous series matrix
MON	motor octane number
MS	mass spectrometry
MTBE	methyl tert-butyl ether
MTHS	molecular type homologous series
MW	molecular weight
N	nitrogen
NetGen	Network Generator
Ni	nickel
NMF	non-negative matrix factorization
NMR	nuclear magnetic resonance
O	oxygen
PDF	probability density function
PINN	physical information neural network
PIONA	paraffins, isoparaffins, olefins, naphthenes, and aromatics
PLS	partial least squares
PNA	paraffin naphthene aromatics
PNN-MS	presampling nearest neighbors–multiple search
PR equation	Peng–Robinson equation
PSO	particle swarm optimization
QSPR	quantitative structure–property relationship
REM	relative entropy maximization
RMG	reaction mechanism generator
RON	research octane number
S	sulfur
SA	simulated annealing algorithm
SARA	saturates, aromatics, resins, asphaltenes
SFEF	supercritical fluid extraction and fractionation
SIHGO	sour imported heavy gas oil
SimDis	simulated distillation
SLO	slurry oil
SM	structural model
SMOG	structural molecular generation
SOHSL-CM	structure-oriented homologous series lumping with cloud model
SOL	structure-oriented lumping
SOLex	extended version of structure-oriented lumping
SQP	subsequent quadratic programming
SR	stochastic reconstruction
SRK equation	Soave–Redlich–Kwong equation
SR-REM	stochastic reconstruction–entropy maximization
SSA	stochastic simulation algorithm
SU	structural unit
SU-BEM	structural unit and bond-electron matrix
TBP	true boiling point
UV-Vis	ultraviolet–visible spectroscopy
V	vanadium
VGO	vacuum gas oil
VPO	vapor pressure osmometry
VR	vacuum residue
**Greek letters**	
α, β and γ	three parameters of the gamma PDF
σ^2^	variance
